# Sociocultural drivers of body image and eating disorder risk in rural Nicaraguan women

**DOI:** 10.1186/s40337-022-00656-0

**Published:** 2022-09-06

**Authors:** T. Thornborrow, E. H. Evans, M. J. Tovee, L. G. Boothroyd

**Affiliations:** 1 School of Psychology, University of Lincoln, Brayford Pool, Lincoln, LN6 7TS Lincolnshire UK; 2 Department of Psychology, Durham University, South Road, Durham, DH1 3LE UK; 3 Department of Psychology, Faculty of Health and Life Sciences, Northumbria University, Newcastle upon Tyne, NE1 8ST UK

**Keywords:** Body dissatisfaction, Body appreciation, Thin-ideal internalisation, Eating attitudes, Eating disorders, Creole, Mestizo, Caribbean, Nicaragua

## Abstract

**Objective:**

Technological and economic globalisation has been suggested as a cause of increasing rates of body dissatisfaction and eating disorders globally, especially as regards the impact of mass media on internalised body ideals. This process is rarely observed in action, however. The current work investigates multiple aspects of body ideals, body image, sociocultural attitudes and eating attitudes in 62 Creole and Mestizo women living in communities at differing stages of technological development on the Caribbean coast of Nicaragua

**Method/results:**

In Study 1, women used 3D avatar software to create their own ‘ideal’ body without the constraints of ready-made stimuli. Analyses of resulting avatars showed that components of the ideal body *shape* (upper and lower body curvaceousness) but not body *size* (body mass) were associated with levels of film and television consumption. In Study 2, women completed measures of variables in the sociocultural model of eating disorder risk. As expected, body dissatisfaction mediated the relationship between internalisation of sociocultural body ideals and pathological eating attitudes. In contrast, body appreciation reduced pathological eating attitudes, via reduced body dissatisfaction. Finally, Study 3 measured sociocultural influences, body image and eating attitudes at 2 or 3 timepoints per woman; body dissatisfaction covaried with pathological eating attitudes across time. Ethnicity varied in its effects across studies.

**Discussion:**

Together these data show that even at early stages of media acculturation, women may show similar patterns of association between sociocultural internalisation, body dissatisfaction and eating disorder risk as in high income nations. However, they also demonstrate unique aspects of this population’s body shape ideals, and the independent protective effect of body appreciation.

**Supplementary Information:**

The online version contains supplementary material available at 10.1186/s40337-022-00656-0.

## Plain English summary

Body dissatisfaction and eating disorders are increasing on a global scale. It has been suggested that increasing access to globalised media maybe one factor in rising risk of eating disorders in low- and middle-income countries. We examined 3-dimensional body ideals, body satisfaction, and eating disorder risk in a population with relatively recent access to mass media. We find that although women in these communities maintain higher body weight ideals, and greater body appreciation, than Western women, those women who are internalising globalised cultural messaging about appearance are at greater risk of body dissatisfaction and, in turn, increased risk of eating disorders. This was true both when comparing between women and looking at how individual women’s attitudes varied over time. These data show that cultural messaging about appearance has implications for body image and eating disorder risk even in populations which do not have a long history of promoting thin ideals.

**Supplementary Information:**

The online version contains supplementary material available at 10.1186/s40337-022-00656-0.

## Background

The prevalence of body dissatisfaction among women in Western populations has reached endemic levels [24, 65]. Body dissatisfaction is both a risk factor for clinical eating disorders [59] and a correlate of other negative outcomes including rigid dieting, low self-esteem, low quality of life and symptoms of anxiety and depression [, 43, 58, 78]. A smaller literature suggests that body and appearance concerns are also increasing in low- and middle-income populations, linked to increasing globalization. For instance, Anderson-Fye [1, 2] has documented nascent body concerns and unhealthy weight control behaviours in girls in rural Belize, while comparing rural and urban samples in South African and Malaysia shows elevated body dissatisfaction, as indexed by women’s ideal versus perceived self in urbanized environments [63]. Similarly, Becker and colleagues’ [5, 6] seminal work in Fiji has shown increasing disordered eating in recent decades, which they connect to changing body ideals through both direct and network-based acculturation to Western media. It is therefore important to examine relationships amongst body ideals, body image, and sociocultural variables that contribute to eating disorder risk in populations undergoing rapid economic and technological development. The current study contributes to this literature by investigating in depth, using multiple approaches, how women in the Caribbean Coast of Nicaragua perceive and feel about their bodies. Furthermore we test key pathways in the sociocultural model [57], examining the relationships between thin-ideal internalization, body dissatisfaction, and pathological eating attitudes, to assess the extent to which Western risk factor patterns are entrenched in this population.

### Media as a source of globalized body ideals

Unrealistic body ideals and pressures to adopt unhealthy weight control behaviours can be transmitted through multiple routes but mostly via family, peers and media (as per the Tripartite Model, [66]). When seeking to understand shifting body ideals and body concerns cross-culturally however, media influence tends to be the focus of interest for researchers. Among Western samples, media exposure is associated with increased body dissatisfaction and disordered eating behaviours among women [27], particularly those considered at high risk [33].

Mass media are highly complicit in constructing, disseminating and perpetuating unrealistic and unachievable appearance standards (or ‘ideals’) in order to sell products and lifestyles and ultimately drive the economy. Western women’s body ideals are predominantly based on extreme slimness (the ‘thin ideal’). It is well established that viewing highly appearance-focused media (e.g., fashion magazines and soap operas) is particularly associated with body dissatisfaction and a drive for thinness [12, 29, 37]. Similarly, experimental studies have also shown that exposure to ‘thin ideal’ media images [7], and objectifying music video content increases body dissatisfaction among women [71], especially among those who have low self-esteem [41].

Few studies of these relationships have been carried out in non-Western populations, but those that exist have identified similar associations between media exposure and body image concerns [13, 40, 63], and between television viewing and dieting in adults [8]. Even among schoolgirls, the onset of media access within a population has been shown to predict thin-ideal internalisation, body dissatisfaction and disordered eating behaviours [5].

### Ethnicity and media influence

Not all women are necessarily equally affected by media globalization. Just as pre-existing body concerns moderate the influence of media within White Western samples, there is some evidence that ethnicity can have a similar moderating effect. For instance, while the gap may be narrowing [51], Black women often have higher body satisfaction than both White women [19, 51] and Asian women [15], and lower levels of eating disorders [84], even when they inhabit the same potential media environment. One explanation of these differences is that individuals in specific ethnic groups may consume mainstream media content to a lesser degree than the predominant demographic (e.g. White Western), resulting in very little internalisation of Western body ideals and subsequently less body dissatisfaction [55]. Alternatively, equal exposure to mainstream beauty ideals may not affect people of all ethnic groups equally. Social comparison theory argues that we tend to make upward social comparisons with others whom we consider not too dissimilar from ourselves [23]. As women presented as ‘attractive’ in mainstream Western media are predominantly White, women of Black ethnic groups may not identify with these constructions of beauty but rather look to other women of colour in the media or in their own social circle as more relevant sources of appearance ideals (see [39], for a recent review of the complexities in the literature around Black women’s body image). In contrast, studies have found that Asian women endorsed Western appearance ideals and had similar levels of body dissatisfaction as White women [15].

Findings are mixed as to whether Latina women experience similar levels of body image concerns as White women (see [81]). Nevertheless, they may be more likely than women of Black ethnicities to regard mainstream standards of beauty as relevant sources for comparison, especially with the increasing visibility in the media of Latina women [80]. However, media representations of *Latinidad* (literally ‘Latin-ness’) are often ‘whitened’ to conform to a Western appearance ideal (i.e., white skin, European facial features and low body weight), meaning Latina women may be just as likely as White women to make appearance comparisons and thus be at risk of body dissatisfaction [15, 36].

Most of these studies examining ethnic differences in sociocultural influences on body image have been carried out in Western contexts, particularly among women in the U.S [10, 11]. At least two studies have shown associations between sociocultural internalisation and body dissatisfaction and eating attitudes in urban, predominantly middle-class adolescents in Latin America (Guatemala: [79], Brazil: [77]. To the best of our knowledge, however, no study thus far has explicitly compared ethnic groups on body image and eating disorder risk in a non-White population with only recent media access.

### Ethnicity and body shape ideals

Differences in women’s experience of their body image may also arise from differences in their body ideals—both in terms of size and shape. In Western populations, the ideal female body has a body mass index (BMI—weight in kg divided by height in metres squared) of about 19–21, the lower end of the normal BMI range [20]. In Korea, even controlling for body size differences, women’s ideal BMI is considerably lower at around 17.9 [36]. There is some evidence to suggest that a slim female body is similarly valued in both Westernized and non-Western urban settings, regardless of ethnic group identity [60]. However, among ethnic groups with a Black African background, the idealised female body tends to be considerably larger, with a BMI of between 25 and 33 kg/m^2^ [8, 35, 69, 73].

In terms of shape, White, Western cultures tend to idealize a ‘thin-but-curvy’ female body: that is, an ‘hourglass’ shape, slender but with relatively large breasts [20, 30]. In contrast, Hispanic and Latina women tend to value a more ‘coca-cola’ shape, which suggests a body that is fuller below the waist than above [54, 81]. Preferences of people from Black ethnic groups also emphasize the lower body shape, often preferring larger buttocks [28, 47, 69].

### The current research

In summary media exposure clearly drives thin-ideal internalization in (some) Western women, and the mass media are potential drivers of thin-ideal dissemination into other cultures, with concomitant implications for body image. However, there is an urgent need for more research to better understand if and how culture and ethnicity intersect in the sociocultural processes that shape body image. For this reason, we took advantage of a period of rapid infrastructural development of a remote region on the Caribbean Coast of Nicaragua to conduct detailed research which considered both sociocultural processes, and the ethnic diversity of the region. When recruiting participants, we specifically focused on Mestizo and Creole women, who comprise two of the four dominant ethnic groups in our fieldsite. In Study 1, we investigated participants’ visual body ideals using interactive avatar software to understand the body size and shape women in this region consider ‘ideal’ and how this related to their television consumption. Study 2 investigated, amongst the same women, the extent to which thin-ideal internalization, via body dissatisfaction, may increase women’s risk of eating disorders, and Study 3 tested these same relationships across time.

### The field site

The Pearl Lagoon Basin is located on the remote Southern Caribbean coast of Nicaragua in Central America. There are few roads in the region and travel between the communities is largely dependent upon water transport in the form of small speedboats or dugout canoes. The terrain around the huge lagoon is dense jungle, interspersed with areas of ‘*potrero*’ where Mestizo farmers have cleared land to graze cattle. However, traditional farming techniques are still used by many indigenous peoples. The local economy is based mainly on fishing and farming (although at the time of writing this is changing). Most communities in the region have between 800 to 1000 inhabitants, although some are considerably smaller. The largest village has a population of about 2000. All communities have at least a primary school, although literacy levels are relatively low and quite variable among both children and adults (field observation). Most people are religious to some extent and all the communities have at least one church, with the majority having several different churches of mainly Christianity-based faiths (e.g., Anglican, Moravian, and Catholic).

The local people are predominantly of Miskitu, Creole, Garifuna, and Mestizo ethnic groups. Miskitu people are an indigenous ethnolinguistic group, Creole and Garifuna people identify mainly as of Black Caribbean or Black African descent. Mestizo people are Spanish speaking Nicaraguans who identify as of predominantly Spanish European descent. Most villages are generally inhabited by people of one ethnic group.

This research recruited women from three locations. Village 1 is a Mestizo community with a population of about 900. At the time of data collection, it had no electricity supply other than a few small solar panels, and therefore very low access to media. Village 2 is a slightly larger Mestizo village that has had generator-supplied electricity to all the community since 2008, and Village 3 is a larger, predominantly Creole community that has had a mains supply electricity for more than a decade. Thus, Villages 2 and 3 had relatively high, regular media access. All participants from Villages 1 and 2 self-identified as Mestizo and all from Village 3 self-identified as Creole. Sampling from these three locations enabled a comparison between 2 ethnically identical groups with different levels of media access, and between two different ethnic groups with similar media access. As there were no magazines, and few people had regular internet access at the time of data collection, overall media exposure could be accurately measured via television (TV) viewing quantity, frequency, and content type.

## Study 1

In Study 1 we investigated whether media consumption was associated with ‘Westernized’ body ideals (i.e. a thin, or ‘thin-but-curvy’ ideal) in terms of both weight and shape, when women completed a task in which they create their own ‘ideal self’ using interactive avatar software. Previously published studies within a similar Nicaraguan population found that television viewing was associated with a preference for a slimmer female body; when viewing photographic stimuli of real European women (men and women); when creating the ‘ideal woman’ (men); and with dieting behaviours among rural women [8]. It was also noted that Mestizo participants favoured slimmer female figures than other ethnic groups [9]. However, beyond Thornborrow et al. [69]’s work with men, all other studies measured participants’ attractiveness judgements based on pre-existing body stimuli depicting European women, so therefore may not fully capture cultural norms for the idealised female body size, or among different ethnic groups. Study 1 therefore used interactive 3D avatar creation software to enable women to create their own ideal body ‘from scratch’ and compared this to their actual body shape and size. Specifically, we were interested in whether television consumption, and participant ethnicity, would influence not just the size of the body they desired, but also the extent to which this body displayed the typical Western idealised features of an hourglass shape with a low body weight (see [30]).

We hypothesized that women of Mestizo ethnicity and thus with a greater degree of European heritage, would favour slimmer bodies—especially in the lower body—than Creole women with a predominantly Black Caribbean heritage. We further hypothesized that women with greater television consumption both at the community and individual level would favour thinner or more ‘Westernized’ body shapes than women with less television access.

### Participants

A total of 62 women (mean age 19.8 years, *S.D* 5.17; range: 14–39) were recruited by word of mouth from Village 1 (N = 19), Village 2 (N = 21) and Village 3 (N = 22) and paid $4 in local currency for their time. Participants chose whether to participate in English or Spanish. All participants from Villages 1 and 2 completed the task in Spanish, and all those from Village 3 completed it in English (with some use of Creole language terms).

### Measures

#### Demographics

Participants reported how many years of schooling they had completed, level of education attained, and total yearly income in U.S dollars or Nicaraguan Cordobás.

#### Media exposure

Participants reported how many hours of television they watched in an average week (TVE), and the frequency with which they watched U.S originating/English language television shows (USTV) and films (USFM), and Latin American originating/Spanish language television shows (SPTV) and films (SPFM) on a scale ranging from 0 (never) to 4 (every day or almost every day). Participants were also asked what kind of content they liked to watch (e.g. *novelas*, music video) and how frequently during a week (0 = none; 1 = < 1 h; 2 = 1–3 h; 3 = > 3 h; 4 = > 5 h; 5 = > 10 h).

#### Actual body size and shape

Height and weight were measured to calculate participants’ Body Mass Index (BMI—weight in kilograms divided by height in metres squared). Bust, waist and hips circumferences were measured to calculate body shape cues waist-to-hip ratio (WHR—waist circumference/hips circumference) and waist-to-bust ratio (WBR—waist/bust). A bust-to-hip ratio (BHR—bust/hips) was calculated to measure relationship of upper to lower body fullness.

#### Perceived current body size

To measure perceived current body size, participants selected an image from the Ten Bodies Scale (TBS—see [68]—a set of 10 images that contain the same figure of a woman of non-white ethnicity but vary in BMI from 15 to 36.5 kg/m^2^. The image set was created using 3D avatar creation software (Daz Studio version 4.6). The body shape changes at different BMI levels are extremely realistic and the 3D rendered stimulus images are high definition and photorealistic. The use of computer-generated imagery (CGI) means that the “identity” of the avatar and its skin texture and proportions remains unchanged across all of the bodies in the set. The only variation in the bodies is due to changing adiposity. This of course would not be possible with the use of photographs of real bodies. To make precise estimates of the BMI of the figures in the images, datasets from the Health Survey for England [45, 46] were used to create calibration curves between waist and hip circumferences and height derived from ∼3500 women in the UK, aged between 18 and 45. The height of the models was then set at 1.6 m allowing the measurement of their waist and hip circumferences, and then comparison with the Health Survey for England calibration curves in order to compute their BMI [17, 18]. The 2D TBS images were printed onto card in full colour and laminated.

#### Ideal body size and shape

Participants created their own ‘bespoke’ ideal body size and shape in the interface of the same ‘3D’ avatar creation software as mentioned above (Daz Studio). When participants are asked to select their ideal from a set of pre-chosen images, even a comparatively large number, the bodies shown inevitably have a limited range of size, shapes and configurations. Therefore it is possible only to say which body the participant prefers *from that set*, and not what their *absolute* ideal is. However, using an interactive 3D modelling program, a participant can create their ideal without these constraints (e.g. [42, 69]). Participants created their ideal body twice, starting each time from a different ‘starter’ body, one with a very low BMI (12.9) and one with a high BMI (35). This was done to minimise possible anchoring effects. See Thornborrow et al. [69] for full details on the Daz programme and methodology.

### Procedure

Participants were interviewed and tested individually in a quiet room. They were informed that their participation was completely voluntary and that they could stop at any time during the task if they did not want to continue. Participants were also told that we were interested in their personal opinions and that there were no ‘right’ or ‘wrong’ answers. Consent was obtained by verbal agreement at the start of the interview. Demographic information was collected verbally and all data were recorded directly onto a laptop. Next, anthropometric measurements were obtained using a digital weighing scale and a tape measure. Participants were weighed and measured without footwear and heavy clothing. They were given the option to measure themselves with guidance if they preferred.

Before beginning the task of creating their ideal body, a ‘trial’ body was opened in the Daz programme to familiarize participants with how the software works, and to demonstrate the full range of body alteration available with each slider. One of the two ‘starter’ bodies was then opened in the interface, and the experimenter operated a set of 20 controls to adjust the figure following the participant’s instructions until she was happy with the ideal body created. The order in which the two starter bodies were presented to participants was counterbalanced. Next, participants were asked to select their perceived current body size from the TBS images.

### Data handling and analysis

Upon completion of all data collection, participants’ ‘ideal’ bodies were re-opened in the Daz programme. The height of the figure was adjusted to participant’s own height before measuring bust, waist, and hip circumferences in centimetres. BMI was calculated using a formula based on real BMI data from the Health Survey for England (as noted above; see also [17, 18]. Ideal lower and upper body shape were measured by calculating the WHR and the WBR respectively. Overall body shape (i.e. hourglass or pear-shaped) was measured by calculating a bust to hip ratio (BHR, bust/hips). A BHR of higher than 1 indicates a ‘top heavy’ shape distribution with a fuller bust and relatively narrower hips, while a BHR of 0.7 indicates a pear-like shape with fuller hips and buttocks than breasts. As participants created two bodies, their body size and shape ideals were calculated by averaging both sets of measurements. ANOVAs and ANCOVAs, controlling for age, comparing across villages (see Table 1) and zero-order correlations were run in SPSS. Finally, a series of multiple regressions were run in R 4.0.3 [49] using RStudio 1.3 [50] for all those media/body ideal associations which were significant in the zero order correlations, with media variables as predictors and body shape ideals as outcomes, with age as a second predictor in each model. During the peer review process, we also ran paired t-tests in base R to compare women’s ideal body size/shape with their actual size/shape. All output can be found in the Additional file 1. A redacted data file (to prevent participant reidentification) and code can be found here: https://osf.io/z8ev2/.

**Table 1:** Unadjusted means for actual, ideal and perceived body size and shape, by village group

		Village 1 (low TV Mestizo)	Village 2 (high TV Mestizo)	Village 3 (high TV Creole)	
Body size	Actual BMI	23.4 (3.35)	26.8 (4.75)	23.3 (5.21)	***F*(2, 58) = 7.281, *p* = 0.002**
	Perceived BMI	22.5 (5.15)	24.0 (4.89)	21.7 (4.27)	F(2, 59) = 1.238, p = 0.297
	Ideal BMI	20.4 (3.12)	21.8 (2.74)	19.8 (2.46)	*F*(2, 59) = 2.477, *p* = 0.093
Body shape	Actual WHR	0.86 (0.05)	0.83 (0.05)	0.76 (0.06)	***F*(2, 58) = 10.074, *p* < 0.0005**
	Actual WBR	0.88 (0.04)	0.85 (0.04)	0.83 (0.04)	*F*(2, 58) = .1.086, *p* = 0.344
	Actual BHR	0.98 (0.04)	0.97 (0.04)	0.91 (0.06)	***F*(2, 59) = 14.563, *p* < 0.0005**
	Ideal WHR	0.70 (0.05)	0.67 (0.06)	0.66 (0.04)	***F*(2, 59) = 4.198, *p* = 0.020**
	Ideal WBR	0.81 (0.05)	0.78 (0.06)	0.75 (0.04)	***F*(2, 59) = 6.470, *p* = 0.003**
	Ideal BHR	0.87 (0.05)	0.86 (0.03)	0.88 (0.04)	*F*(2, 59) = 1.532, *p* = 0.225
Body image and eating behaviours	BAS	63.00 (3.28)	58.95 (7.35)	58.59 (5.37)	***F*(2, 59) = 3.726, *p* = 0.030**
	BSQ-8c	12.37 (4.46)	17.14 (8.76)	17.55 (8.45)	*F*(2,59) = 3.069, *p* = 0.054
	Dieting % (N)	21.7 (5)	43.5 (10)	34.8 (8)	χ^2^ = 2.94, *df* = 4, *p* = 0.559
	EAT-26	3.74 (3.75)	6.57 (6.96)	5.64 (5.98)	F(2, 37.8) = 1.624, *p* = 0.211
	SATAQ	34.4 (15.54)	56.7 (17.28)	43.7 (15.91)	***F*(2, 59) = 9.464, *p* < 0.0005**

*BMI* Body mass index (weight in kilograms divided by height in metres squared); *WHR* Waist-to-hip ratio (waist circumference divided by hips circumference); *WBR* Waist-to-bust ratio (waist circumference divided by bust circumference); *BHR* Bust-to-hip ratio (bust circumference divided by hips circumference). Significant (*p* < 0.05) results noted in bold

### Results

**Fig. 1:**
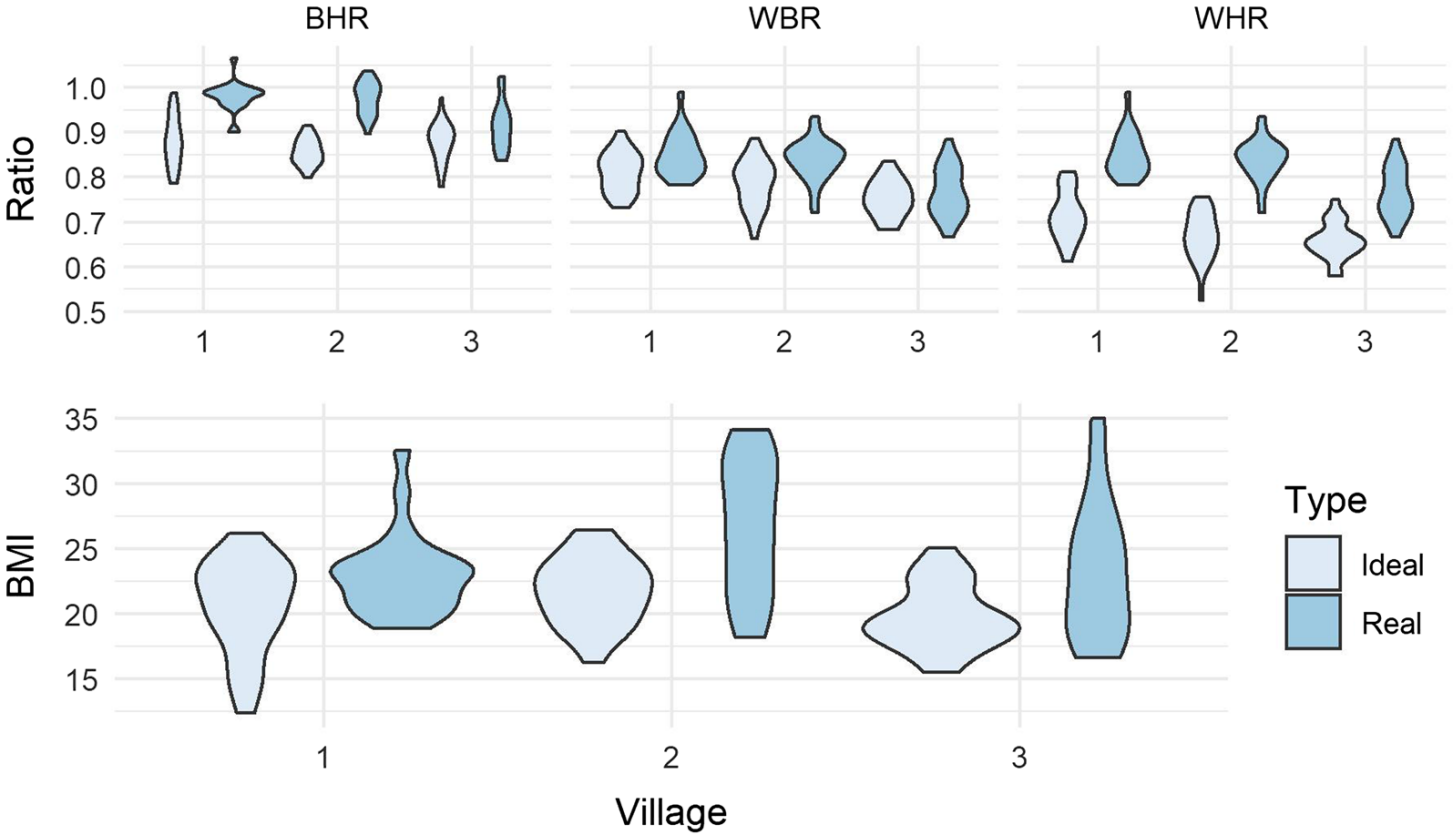
Distribution of actual and ideal (Daz) body size and shape proportions in each village (Village 1 = low TV Mestizo, Village 2 = High TV Mestizo, Village 3 = High TV Creole)

Means for age, education, yearly income, and media consumption of participants for all three villages are given in Additional file 2: Table S1. There were some demographic differences; Village 1 sample was significantly older than samples from Villages 2 and 3, *F*(2, 59) = 18.532, *p* < 0.0001, Games Howell *p*s < 0.01, who did not differ from each other (*p* > 0.05). The Village 3 sample was more educated than Village 2, which was more educated than Village 1, *F*(2, 59) = 35.823, *p* < 0.0001, post hoc *p*s < 0.01. There were no significant differences in income. We were able to confirm that the high TV villages (2 and 3) consumed more media than Village 1; in general, Village 3 consumed the most on all measures except watching telenovelas which was more common amongst women in Village 2. Comparing women’s ideal and actual body size/shape, we find that paired t-tests show significant differences at the whole-sample level for BMI and all three body proportions; as shown in Table 1 and Fig. 1, women desired smaller bodies, with smaller waists and larger hips than their own.

#### Group differences

See Table 1 for means of participants’ actual body size and shape, and perceived body size for all village samples. As actual BMI, actual WHR, and actual WBR were significantly correlated with age, and women generally become heavier and less curvaceous with age [83] analysis of mean differences was carried out using ANCOVA with village as a fixed factor and age as a covariate. Women’s actual BMI differed significantly between villages, such that the (Mestizo) women in Village 1 were significantly slimmer than their compatriots in Village 2, *p* = 0.001, while the (Creole) women in Village 3 did not differ from either (*p*s > 0.05). Women’s body shapes also differed between villages; Creole women in Village 3 had much curvier lower bodies with larger hips relative to both their waists (WHR) and busts (HBR) than the Mestizo women in Villages 1 and 2 (*p*s < 0.001) who did not differ from each other (*ps* > 0.05). Although perceived BMI was highly correlated with women’s actual BMI (*r* = 0.76, *n* = 62, *p* < 0.0005), the groups did not differ.

There were no significant differences in women’s ideal BMI across villages, however there were for both ideal WHR and WBR. For both body shape variables, Village 3 means were significantly lower (thus indicating a curvier ideal body shape) than those of Village 1. Village 2 means were intermediate but not significantly different from either (*p*s > 0.05).

Pearson’s correlations examining associations between ideal body size and shape variables and media viewing variables are shown in Table 2. As media viewing variables USFM (US/English-language film viewing frequency) and SPFM (Spanish-language film viewing frequency were highly correlated (*r* > 0.8) they were collapsed into one variable FILM. Ideal WBR was significantly correlated with all measures of media consumption in the predicted direction, such that a curvier upper body preference (smaller waist relative to bust) was associated with more media viewing. Similarly, those who reported watching English language television more frequently created figures with a smaller waist relative to hips. When age was added as a covariate in the multiple regression models for those pairs of variables with significant bivariate correlations, the associations between frequency of watching US/English language television continued to predict body shape preferences (WHR: *B* = -0.013, *SE* = 0.005, *p* < 0.05; WBR: *B* = − 0.011, *SE* = 0.005, *p* < 0.05), but other associations did not (*p*s > 0.05; see Additional file 1: Sect. 1.3 for full results).

**Table 2:** Pearson’s correlations between ideal body size and shape and media viewing variables

	BMI	WHR	WBR	BHR
TVE	0.013	− 0.225	− 0.283*	0.053
SPTV	0.054	− 0.208	− 0.257*	0.034
USTV	− 0.050	− 0.297*	− 0.338**	0.021
FILM	− 0.117	− 0.130	− 0.280*	0.211

*p < 0.05, **p < 0.01. *BMI* Body mass index (weight in kilograms divided by height in metres squared); *WHR* Waist-to-hip ratio (waist circumference divided by hips circumference); *WBR* Waist-to-bust ratio (waist circumference divided by bust circumference); *BHR* Bust-to-hip ratio (bust circumference divided by hips circumference). *TVE* Average hours of TV viewing per week; *SPTV* Viewing frequency of Latin American originating/Spanish language television shows; viewing frequency of *USTV* U.S originating/English language television shows; *FILM* Combined viewing frequency of U.S originating/English language and Latin American originating/Spanish language films

### Interim discussion

Overall, these results suggest that Creole women in a high media environment, and women in this sample who consume English-language (i.e. ‘Western’) television more regularly, favour a curvier body shape with a smaller waist compared to hips and busts, relative to other women, even Mestizo women with a low media intake. These differences cannot be simply attributed to actual differences in women’s own bodies as the Creole women had larger hips but not narrower waists (relative to busts) than Mestizo women. These results therefore suggest body shape preferences may be culturally variable in this setting.

On the other hand, we did not replicate results from our studies using different methodologies which found that media consumption affected body *size* preferences [8, 9, 35]. Those studies, which had larger samples of both women and men, gave participants real or artificial-but-lifelike bodies which had either been preselected or created to vary in body mass. Furthermore, those stimuli showed, or were based on, body shape variation patterns in White British women, who tend to deposit additional body fat differently to Creole or Latina women. Therefore, participants indicated their preferences within a limited and potentially less ethnically typical range of body shapes. In the present study, when women created their own ‘ideal’ body without such constraint, media consumption/access did not impact overall slimness so much as reducing waist size. This was particularly the case for participants who reported consuming US media specifically, supporting evidence of a ‘curvy-but-thin’ ideal body [30]. We note, however, that women across the sample created bodies which were generally of lower BMI than themselves (insofar as projection from 3D stimuli can relate to real body mass/composition). In fact, while the mean ‘ideal self’ in each community was within the ‘healthy’ range as defined by medical bodies, they were at the lower end of this range and in the Creole community in particular, would not have offered resilience against periodic food shortages. As we have previously noted (see [35]), this population is subject to periodic nutritional stress, especially those communities (such as Village 3) which are partially reliant on fish levels in the Lagoon. As such, there is no ‘buffer’ for future reductions in women’s target/ideal body size in this population if it should affect behaviour.

## Study 2

In Study 2, we employed Stice’s sociocultural model of disordered eating to explore the relationship between media influence, body dissatisfaction, and disordered eating behaviours [56, 57]. The model posits that culturally constructed ideals, including those transmitted via the media, may become internalised by some individuals to the point that they measure themselves in relation to that ideal. Subsequently they will experience satisfaction or (more usually) dissatisfaction, depending on how congruent they consider themselves to that ideal [70] which will increase the risk of pathological eating attitudes, via unhealthy dieting and negative affect. Studies among both Western and Latin American young populations have found such models useful in explaining the role of media exposure in body image outcomes [22, 40, 77, 79].

While Study 1 focused on women’s idealised body shapes, Study 2 focused on whether they internalised media ideals more broadly, and whether they also experienced concomitant body dissatisfaction and eating disorder symptoms. The same participants as in Study 1 completed questionnaire measures of these constructs. We first hypothesised that women in high media environments would show greater thin-ideal internalisation, reduced body appreciation, more body dissatisfaction, and score higher on eating disorder symptoms. We further hypothesised that thin-ideal internalisation would predict disordered eating via body image.

### Measures

#### Media beliefs

A modified version of the Sociocultural Attitudes Toward Appearance Questionnaire -3 [67] was administered to assess participants’ attitudes towards, and internalisation of, media messages relating to physical appearance. Negatively keyed items were removed following preliminary testing as the negative wording caused confusion for participants in this population. Items that mentioned magazines were also removed, as magazines are not generally available in the region. Items that related to magazines and television were modified to mention television only. Some local Creole terms were added to aid participants’ understanding (e.g. ‘I feel pressure from TV to be thin (meagre)’). A Cronbach’s alpha of 0.91 demonstrated a high level of internal consistency for SATAQ total scores.

#### Body image

The Body Appreciation Scale [4] was administered to establish participants’ general body appreciation. The 13-item measure was originally developed and validated for use among women in the U.S, and later for use among men [75]. The Spanish version was developed and validated with a sample of Spanish male and female adolescents [38]. It has also been used to measure body appreciation in non-Western and non-White populations, although some studies suggest it may not have the same one-factor structure in all populations [19, 61, 64]. Scores on the BAS scale demonstrated a good level of internal consistency, with a Cronbach’s alpha of 0.84.

A short version of the Body Shape Questionnaire [16] was administered to identify body dissatisfaction. The BSQ-8c [21] performs well in both English and Spanish speaking Western populations [48, 82]. However, one study with a sample of Colombian adolescents found that the original BSQ had a two-factor structure, suggesting that Latin Americans may interpret or experience body dissatisfaction differently than Spanish-speaking groups from the U.S or Europe [14]. Here, BSQ scores had good internal consistency (Cronbach’s alpha 0.80).

#### Eating disorder symptoms

The Eating Attitudes Test EAT-26 [25] was employed to assess attitudes towards eating and behaviours around food that correspond to the symptoms of subclinical and clinical eating disorders. The measure has been validated for use in screening for disordered eating behaviours among non-clinical samples and has been used as an outcome measure in both Western [12, 31] and non-Western populations [2, 5, 13]. Cronbach’s alpha for EAT-26 scores was good at 0.84.

### Procedure

After completing the ideal body task for Study 1, participants responded to the psychometric measures in the following order: BAS, SATAQ-3, BSQ-8c, EAT-26. The participant was given the option of reading the items aloud themselves, or the experimenter reading them aloud. Participants indicated their responses on laminated copies of the measures, and the experimenter entered their verbal responses into the laptop at the same time. This procedure was implemented to ensure that participants with varying degrees of literacy understood the items and to aid in task engagement.

### Data analysis

All questionnaire scores were calculated as the sum of all items (using the relevant scoring protocol for items). As in Study 1, ANCOVAs comparing questionnaire scores across villages were run in SPSS with age as a covariate. All other analyses were completed in R. Initial group comparisons used total SATAQ scores; however thereafter the scores for individual subscales were used in order to focus on internalisation specifically. Correlations between all variables, and participant BMI and age were run, followed by mediation models to test the core components of the sociocultural model (that internalisation should predict body dissatisfaction which then predicts eating attitudes.) As shown below, age was not associated at zero order with internalisation, dissatisfaction or eating attitude scores, so was not included as a covariate. Following exploratory modelling, a path model was run in *Lavaan* [52] to demonstrate one potential set of relationships between variables including body appreciation as well as body dissatisfaction. All code and output is available in the Additional file 1.

### Results and discussion

Table 3 shows means and standard deviations for questionnaire measures by village sample. Significant differences were found between groups’ mean BAS and SATAQ scores. Tukey post hoc tests showed that Village 3 women had significantly lower body appreciation than those of Village 1 (*p* < 0.05). Village 2 means did not differ from either (*p*s > 0.05). Mestizo women in high-media Village 2 showed greatest thin-ideal internalization as measured by global SATAQ scores, followed by Creole women in high-media Village 3, with the Mestizo women in low-media Village 1 showing the lowest scores.

**Table 3:** Unadjusted body image and eating attitude questionnaire scores by village

	Village 1 (low TV Mestizo)	Village 2 (high TV Mestizo)	Village 3 (high TV Creole)	
BAS	63.00 (3.28)	58.95 (7.35)	58.59 (5.37)	***F*(2, 59) = 3.726, *p* = 0.030**
BSQ-8c	12.37 (4.46)	17.14 (8.76)	17.55 (8.45)	*F*(2,59) = 3.069, *p* = 0.054
EAT-26	3.74 (3.75)	6.57 (6.96)	5.64 (5.98)	F(2, 37.8) = 1.624, *p* = 0.211
SATAQ (total)	34.4 (15.54)	56.7 (17.28)	43.7 (15.91)	***F*(2, 59) = 9.464, *p* < 0.0005**

Significant results noted in bold

**Fig. 2:**
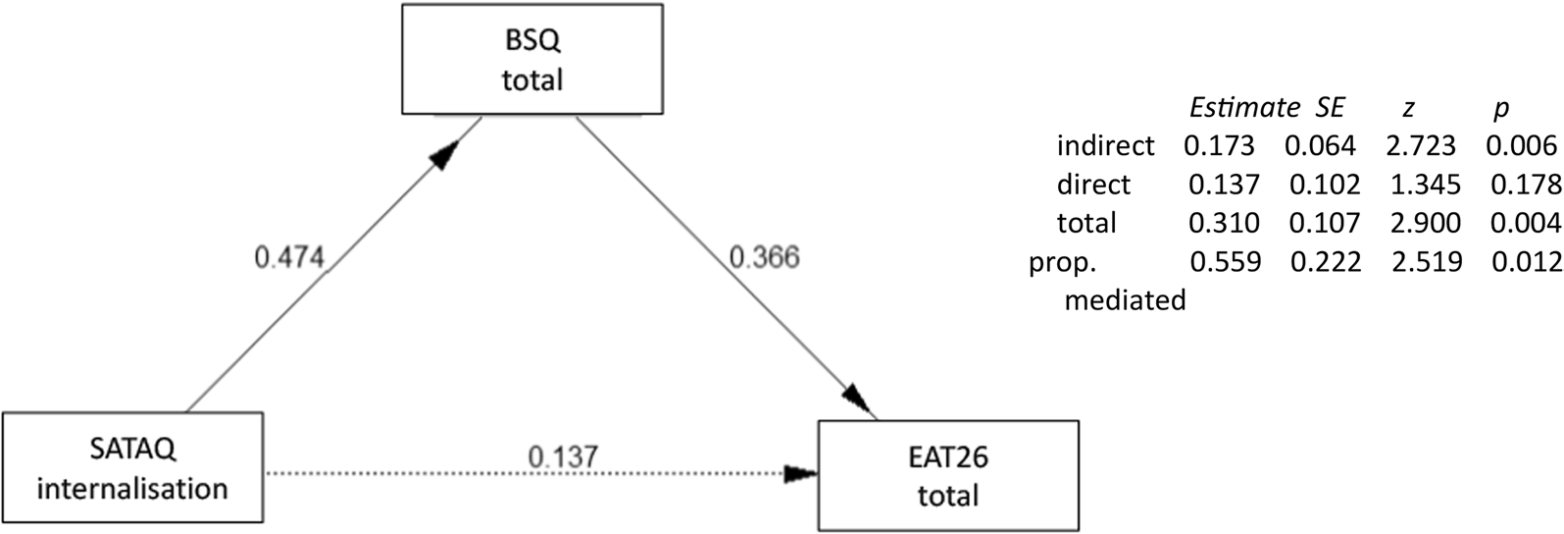
Mediation analysis of SATAQ internalization on EAT26 scores via BSQ*.* Diagram shows the regression estimates for each path in the mediated model

Table 4 presents Pearson’s correlations between EAT, SATAQ, BSQ, BAS, and participant’s own BMI for the whole sample (see Additional file 2: Table S2 for correlations within ethnic groups). As predicted, higher media appearance internalization (SATAQ) in general—and both internalization and perceived pressures when looking at subscales—were significantly associated with lower body appreciation. Supporting Stice’s sociocultural model of disordered eating [56], there were significant positive associations between eating disorder symptoms and levels of both media appearance internalisation and shape concerns. Mediation models (using the PROCESSR package—[44] were used to assess whether thin-ideal internalization (using specifically the internalization subscale of SATAQ) acted on eating disorder symptoms via body dissatisfaction (BSQ-8c). As shown in Fig. 2, we found a significant total effect of SATAQ-internalization on eating disorder symptoms, with significant mediation through shape concerns (see Additional file 1: For full model summary, and additional regression models demonstrating this pattern held with age included as a covariate).

**Table 4:** Pearson’s correlations between disordered eating behaviours (EAT-26) and predictors based on Stice’s sociocultural model, media ideal internalisation (SATAQ), general body satisfaction (BAS), body dissatisfaction (BSQ-8c) and own body mass index

	1	2	3	4	5	6	7	8	9
1. BMI									
2. BAS	0.02								
3. BSQ	0.48**	− 0.42**							
4. SATAQ total	0.17	− 0.40**	0.41**						
5. SATAQ -internalisation	0.08	− 0.34**	0.38**	0.87**					
6. SATAQ -pressure	0.22	− 0.41**	0.46**	0.91**	0.74**				
7. SATAQ -information	0.10	− 0.25*	0.23	0.77**	0.53**	0.54**			
8. EAT-26	0.40**	− 0.29*	0.54**	0.33**	0.33**	0.39**	0.20		
9. TVE	0.16	− 0.22	0.29*	0.10	0.07	0.06	0.23	0.21	
10. Age	0.22	0.30*	− 0.11	− 0.34**	− 0.19	− 0.24*	− 0.47**	− 0.03	− 0.37**

*p < 0.05; **p < 0.01. *BMI* Participants’ own body mass index; *BAS* body appreciation scale; *BSQ* Body shape questionnaire version 8-c; *SATAQ* Sociocultural attitudes towards appearance questionniare; *EAT-26* Eating attitudes test 26-item version; *TVE* Average hours of TV viewing per week; *Age* Participant age in years

**Fig. 3:**
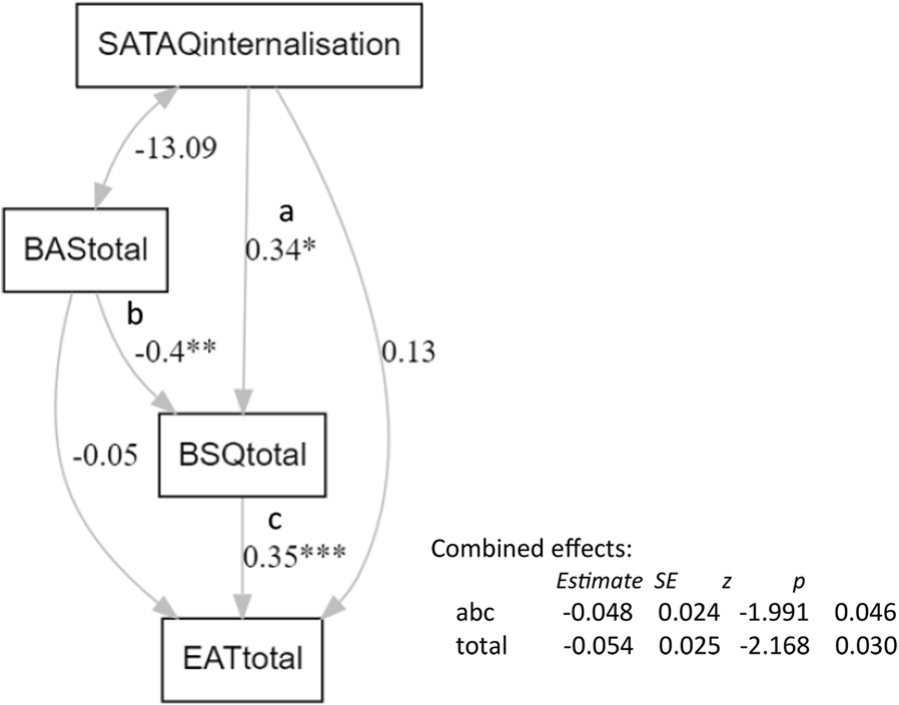
Structural equation model with BSQ as a mediator between both BAS and SATAQ internalisation, and EAT26 scores

Further exploratory models tested whether body appreciation (i) could be substituted for body dissatisfaction, or (ii) is a protective factor preceding body dissatisfaction. Initial conceptualisations of body appreciation framed it as the inverse of body dissatisfaction, but more recent conceptual expositions (e.g. [76] frame it as a broader construct: for example, it is possible to have low levels of both body appreciation and body dissatisfaction). These models favoured the latter interpretation. Specifically, including BAS as the mediator instead of BSQ resulted in no significant mediation (proportion mediated = 0.21, z = 1.38, *p* = 0.17) while substituting SATAQ-internalisation with BAS as predictor did show significant mediation between BAS and EAT26 scores through BSQ (proportion mediated = 0.72, z = 2.41, *p* = 0.016; see Additional file 1: For full results and additional models). A full model was therefore constructed in *Lavaan* with both internalization and body appreciation feeding into body dissatisfaction, with indirect effects on eating attitudes. Results are shown in Fig. 3 and demonstrate a significant combined path between BAS/SATAQ internalisation scores, BSQ and EAT26 scores. Full results for the model are found in the Additional file 1.

#### Interim discussion

In summary, therefore, these cross-sectional analyses suggest that within a rapidly developing population, women in higher-media environments experience reduced positive body image/body appreciation, regardless of ethnicity. Higher media environments were also associated with greater thin-ideal internalisation, although this was more the case for the Mestizo women than the Creole women—despite the Creole participants actually watching more television. This offers support to the argument that women of Black ethnic groups may be less affected by media ideals than women of other ethnic groups [15]. This finding could also be explained by ethnic differences in actual content viewed. Relative to Creole women, Mestizo women in Village 2 reported watching more telenovelas, which have strong focus on stereotypically slim, attractive female characters. Finally, the fact that disordered eating attitudes were predicted by media internalisation and body dissatisfaction confirms findings from previous studies in urban Latin American populations [13]: [79] and adds support to the utility of a sociocultural model of disordered eating [56] outside of a Western context, even in rural, less globally acculturated communities. We note, however, that in this cultural context, body appreciation may positively counter effects of thin-ideal internalization and thus reduce women’s dissatisfaction with their body (as the BSQ measures).

## Study 3

The aim of Study 3 was to assess whether the cross-sectional observations in Study 2 held when considering variation within women over time. This study had been planned as a way of capturing the effects of electrification and an anticipated accompanying increase in media consumption in Village 1. As discussed elsewhere [9] the electrification project was delayed, and re-testing of participants was challenging. Instead, therefore, we simply considered covariation between components of the sociocultural model over time within our sample as a whole. If the sociocultural model holds true, then as women experience more (or less) sociocultural appearance pressures and internalization, so too would they experience more (or less) body dissatisfaction and increased (or decreased) pathological eating attitudes.

### Methods

Of those participants in Study 1 and 2, 34 were re-tested on a second and/or third visit. Original data were collected between late 2014 and 2015, with follow ups between late 2016 and 2017 and finally in 2019. In total 12 women from Village 3 took part on two occasions, 15 women in Village 2 took part more than once (9 twice, 6 three times) and 7 women from Village 1 (5 twice, 2 three times). As such participants in Study 3 were predominantly those from higher media environments. Participants completed all measures from Studies 1 and 2 on each visit. However, in the current study we focus on their questionnaire measure responses.

### Data analysis

To assess covariance across time within individuals, data were entered into a hierarchical series of mixed effect models in the *lme4* package in *R*, where participant was entered as a random factor. Initial models tested covariation between SATAQ internalisation scores and body dissatisfaction. To assess the mediation component of the results of Study 2, further models then tested effects on eating disorder symptoms, with internalisation and body dissatisfaction entered sequentially. Finally, timepoint (as a categorical predictor) was added in the final model to determine whether time also had a directional effect on scores.

### Results and interim discussion

The main models are summarized in Table 5. Results confirmed that internalization and body dissatisfaction covaried over time, such that women were more likely to report being unhappy with their bodies at points in time where they also expressed stronger internalisation of the thin ideal. The direct relationship between SATAQ scores and EAT26 scores did not reach significance in these models, however, BSQ scores continued to predict EAT26 scores as in the within-time mediation models above. Finally, when time point was added we saw there was also an increase in eating disorder risk amongst our repeat-participants at Time 3. Time 3 EAT26 scores were, on average, 3.4 points higher than at Time 1, supporting the concern that eating disorder risks are increasing in rapidly developing populations.

**Table 5:** Mixed effect linear models showing co-variance of internalisation, body dissatisfaction and eating attitudes over time within women

	Outcome: BSQ		Outcome: Eat 26					
	*1*		*2*		*3*		*4*	
	*B*	*SE*	*B*	*SE*	*B*	*SE*	*B*	*SE*
Constant	11.502**	(− 1.821)	3.842*	(− 1.656)	− 1.328	(− 1.791)	− 2.354	(− 1.867)
SATAQ-internalisation	0.407**	(− 0.141)	0.236	(− 0.127)	0.054	(− 0.117)	0.05	(− 0.112)
BSQ					0.449**	(− 0.092)	0.428**	(− 0.088)
Time (ref: Time 1)								
Time 2							− 0.077	(− 1.72)
Time 3							3.443**	(− 1.263)
Observations	76		76		76		76	
Log Likelihood	− 258.344		− 250.923		− 241.948		− 235.37	
AIC	524.687		509.847		493.896		484.74	
BIC	534.01		519.17		505.55		501.055	

Model 1 shows the univariate association between internalisation and body dissatisfaction, while Models 2–4 show the association of internalisation, body dissatisfaction and time on eating attitudes

*p < 0.05; **p < 0.01

These results provide further evidence that the sociocultural model of eating disorder risk is relevant in the context of a region undergoing rapid technological development. Although we lacked power for lagged analyses (e.g. whether internalisation at Time 1 predicted body dissatisfaction at Time 2), these factors nevertheless covaried together within individuals as the sociocultural model would predict.

## General discussion

Data from Western countries have long suggested that visual media play a role in promoting and maintaining a thin-ideal appearance standard, with subsequent impacts on body image and eating disorder risk especially but not only among young women. However, it was less clear to what extent this would apply in populations where access to the mass media is relatively novel. Furthermore, data from US populations in particular raised questions regarding the extent to which women of non-White and especially Black ethnicities would show the same patterns observed in White women. This research therefore aimed to investigate the influence of media on women’s body ideals and body image in a technologically rapidly developing non-Western, non-White population. Due to the unique conditions in our field site, we were able to compare body image outcomes among groups of women with significantly different levels of media exposure. Furthermore, through repeat visits we were able to follow some of these women over a period of 2–4 years.

Study 1 used interactive 3D avatar software to investigate women’s body size and shape ideals in a manner which did not constrain their responses to pre-set dimensions. Measuring the proportions and overall sizes of those bodies showed no clear differences regarding ideal body size but did show that women from communities with more media access tended to create bodies with smaller waists relative to hips and busts (i.e. curvier body shapes), particularly the Creole women. Women who reported watching American television more regularly also created bodies with a curvier shape.

Study 2 found that overall our participants had a generally positive body image (relative to similarly diverse samples in the UK; see e.g. Swami et al. [62]). However, signs of sociocultural pressures impacting body esteem and eating attitudes were present and in Study 3 we saw a significant increase in disordered eating attitude scores at the third timepoint relative to baseline (although note, that increase remained even when body dissatisfaction was included in the model so is not just a result of increasing body dissatisfaction over time). Although individual levels of reported television consumption did not associate with many attitudinal measures of body concerns, the women in high-media communities had stronger internalisation of cultural appearance pressures and lower body appreciation. Furthermore, internalisation of the thin-ideal in this population appeared to increase eating disorder risk via body dissatisfaction—both when looking across individuals (Study 2) and when looking at variation over time within women (Study 3). Body appreciation, on the other hand, seemed to act as a protective factor against body dissatisfaction by providing an additive (rather than interacting) counter to internalisation.

An additional finding of note is that women in the low-media village created ‘ideal’ bodies which were just as slim as those created by women in the high media villages. Previous data we collected found a slightly differing pattern of preferences: Residents in the same low-media village favoured slightly larger bodies than those in the high-media Mestizo village. However, regardless of location and media exposure Mestizos in that previous study preferred considerably slimmer bodies than participants of Black and Amerindian ethnicities (e.g. in one community, women with a BMI of over 30 kg/m^2^ were rated as most attractive; [9]. This is consistent with the suggestion that the ‘traditional’ appearance ideal among some ethnic groups is already based on a slim body type irrespective of media exposure [60]. Another potential influencing factor on body ideals are local cultural constructions of gender [34]. Certainly, the body size ideals of men and women in the rural Mestizo communities in particular appear to be based on stereotypically dimorphic constructions of gender; small, slender bodies for women and larger, heavier bodies for men [68]. Some authors have suggested that in some cultural contexts, traditional gender roles (and thus stereotypical appearance norms for men and women) may persist in spite of economic/technological development [34].

As we predicted, Mestizo women in high TV Village 2 had much greater internalisation of media appearance ideals, measured by SATAQ, than Mestizo women in low TV Village 1. Furthermore, Creole women had significantly lower internalization of media’s appearance standards than Mestizo women, reflecting findings from studies in the U.S that sampled Black and White women [15]. Creole women’s lower internalisation could result from watching less appearance-focused content in general, or from not being influenced by it to the same degree as Mestizo women. According to social comparison theory [23], comparisons are made with individuals who are considered approximately ‘comparable’, therefore Black Creole women may identify less with women appearing on TV in Nicaragua because the vast majority of them are not Black. In the U.S, while less than 20% of the people that appear on prime-time TV shows are Black, the figure generally reflects the percentage of Black Americans in the population at national level [74]. In our rural Nicaraguan field site, however, the percentage of Black people in the local population is much higher than the percentage seen on television, so potentially there is less opportunity for meaningful appearance comparisons for Black women than for Mestizo women.

The contribution of ethnicity also provides a possible explanation for the differences we found in our Creole and Mestizo women’s eating behaviours. Previous evidence indeed suggests that Black women, especially those with a strong ethnic identity, are less likely to experience dissatisfaction with their weight because they are more accepting of a range of body sizes [32]. Additionally, a full and curvaceous body shape, which is not dependent purely upon a certain body weight or BMI, is often more central to Black women’s ideal body type [26, 47]. Therefore, women of Black ethnic groups may be less likely to modify their eating behaviour in service of having a lower body weight. In combination these findings suggest that Black ethnic identity can be a protective factor against eating disorder symptoms [19] and can predict a healthier body image [55].

We found further evidence that body size or weight was not the most salient aspect of women’s ideal body in this sample. Although media exposure was associated with women’s ideal body shape and lower levels of body appreciation, it was not associated with women’s actual BMI or their ideal BMI. Moreover, although our analysis confirmed previous studies [53] and added support to the validity of using a sociocultural model of disordered eating [56] outside of a Western population, the BSQ taps strongly into body *shape* concerns, rather than specifically weight dissatisfaction. As such, it may be a particularly useful measure for predicting disordered eating among Nicaraguan women where shape concerns may exceed size concerns. This is further supported by the fact that general body appreciation seemed to act as an (inverse) antecedent or protective factor in regard to body dissatisfaction, rather than its inverse [3, 72]. In this context, body appreciation is usually defined as more than simple (lack of) concerns about the body’s surface appearance, but also includes features such as the body’s capabilities, health and well-being, and is regarded as a separate construct to, rather than the obverse of, body dissatisfaction [4]. These results reinforce the argument that body appreciation is an important potential route for intervention to prevent body dissatisfaction.

Furthermore, our findings suggest that, in this population, a rather more ‘upstream trigger’ for eating concerns—belief in media’s appearance standards (SATAQ scores)—may be a better measure of media impact on women’s body image and eating behaviours than simply television viewing. Watching television per se is not necessarily the same as being exposed to specifically appearance-focused visual content: someone watching *novelas* or music videos for four hours every day is more likely to be exposed to, and potentially internalize, media messages about appearance ideals than someone who watches four hours of news programmes, for example.

In summary, our findings suggest that an idealised body size or weight may not be the most salient aspect of women’s body ideals and body image in this population. However, media internalisation does appear to be associated with other idealised body components such as shape (i.e. importance of curviness and ‘booty-ness’), affecting how satisfied these women feel with their bodies in general, and potentially increasing their risk of developing pathological eating attitudes.

It is important to note however, that this research involved a small sample which necessarily limits how broadly we can apply the results. Previous research at our field site with partially overlapping communities showed a significant effect of television on body size preferences (using related but slightly different methods)—both in a cross-sectional sample of approximately 300 individuals, and an experimental study with 80 participants [9]. The current study was much smaller to allow us to use the participant-led 3D avatar creation task which took considerably longer than other methods of data collection. However, the direction of means does not suggest that a larger sample would have yielded matching results on body size preferences to our previous studies. Additionally, the lack of other ethnicities represented in low media communities in the current study may have obscured effects on body size preferences that we could not observe in Mestizos. Further work using participant-led methods such as our Daz software paradigm among diverse ethnic groups is clearly warranted.

Our relatively small sample also means that we should treat the modelling in Studies 2 and 3 with some caution. The fact that our results converge with those from much larger Western datasets and urban Latin American samples as regards the sociocultural model [11, 77, 79] gives some confidence in the results. The final model in Study 2, however, was exploratory and should primarily form a basis for further research on body appreciation and thin ideal internalisation as independent precursors of good (or bad) body esteem/concerns and eating pathology risk in ethnically and culturally diverse settings.

Finally, although the current research partly employed participant-led ‘objective’ construction of a hypothetical body ideal, this may still not incorporate all aspects of appearance concern or body image experienced by these women. Our published qualitative work with men in this region demonstrates, for instance, that movement is very important in how men think about women’s bodies [69]. Similarly, other factors may be important in how women construct their own body cognitions, for example skin tone and quality and hair (see e.g. [39], for how these may be particularly important for Black women).

## Conclusions

In conclusion, this study provides an important in-depth study of body ideals and body image, and its precursors, and consequences for eating attitudes, in three small rural communities in Nicaragua. Our findings raise concerns that the rapid infrastructural and technological development the region is experiencing may have the potential to contribute to increasing levels of poorer body image and risk of developing eating disorders. Further work investigating both body ideals and sociocultural impacts on body image in other similarly developing populations is strongly needed.

## Supplementary Information

**Additional file 1:** Full analysis output.

**Additional file 2: Table S1.** Means and standard deviations for sample characteristics, and media viewing variables by village group. **Table S2.** Pearson’s correlations between disordered eating behaviours (EAT) and predictors based on Stice’s sociocultural model, media internalisation (SATAQ), general body satisfaction (BAS), body shape concerns (BSQ) and own BMI for Mestizo and Creole women.

## Data Availability

Data have been redacted to prevent re-identification and are available with analysis script at https://osf.io/z8ev2/.
