# Supplementary material for: Sociocultural drivers of body image and eating disorder risk in rural Nicaraguan women
Source: J Eat Disord. 2022 Sep 6;10:133. doi: 10.1186/s40337-022-00656-0 (PMC9450464; doi:10.1186/s40337-022-00656-0)
Supplement: Supplementary file 1 — Additional file 1: Full analysis output. [file 40337_2022_656_MOESM1_ESM.html]

Thornborrow et al Sociocultural influences on body image and eating attitudes in Nicaragua - code and output


# Thornborrow et al Sociocultural influences on body image and eating attitudes in Nicaragua - code and output

# 1. Study 1 - Body shape ideals

## 1.1 Correlations

```
corlist.s1 <- select(dazdat1, c(BMI, TVhrs, Age, daz_BMI, Discrep, daz_WHR, daz_WBR, daz_BHR))
apa.cor.table(corlist.s1, show.conf.interval=F, filename = "DAZcors_s1.doc")
```

```
## The ability to suppress reporting of reporting confidence intervals has been deprecated in this version.
## The function argument show.conf.interval will be removed in a later version.
```

```
## 
## 
## Means, standard deviations, and correlations with confidence intervals
##  
## 
##   Variable   M     SD    1           2            3           4           
##   1. BMI     24.44 4.83                                                   
##                                                                           
##   2. TVhrs   10.98 10.62 .16                                              
##                          [-.08, .38]                                      
##                                                                           
##   3. Age     19.68 4.96  .22         -.37**                               
##                          [-.02, .43] [-.55, -.15]                         
##                                                                           
##   4. daz_BMI 20.76 3.01  .46**       .01          .16                     
##                          [.25, .62]  [-.22, .24]  [-.07, .38]             
##                                                                           
##   5. Discrep 1.87  5.21  -.03        .15          -.11        -.52**      
##                          [-.27, .21] [-.10, .37]  [-.34, .13] [-.67, -.32]
##                                                                           
##   6. daz_WHR 0.68  0.06  .00         -.25*        .20         .19         
##                          [-.23, .24] [-.45, -.01] [-.04, .41] [-.05, .40] 
##                                                                           
##   7. daz_WBR 0.78  0.05  .12         -.30*        .31**       .44**       
##                          [-.12, .34] [-.50, -.07] [.08, .50]  [.23, .61]  
##                                                                           
##   8. daz_BHR 0.87  0.05  -.13        .03          -.12        -.28*       
##                          [-.35, .11] [-.21, .26]  [-.34, .12] [-.48, -.05]
##                                                                           
##   5            6          7          
##                                      
##                                      
##                                      
##                                      
##                                      
##                                      
##                                      
##                                      
##                                      
##                                      
##                                      
##                                      
##                                      
##                                      
##   -.13                               
##   [-.35, .12]                        
##                                      
##   -.24         .76**                 
##   [-.45, -.00] [.64, .85]            
##                                      
##   .11          .56**      -.11       
##   [-.13, .34]  [.37, .70] [-.33, .13]
##                                      
## 
## Note. M and SD are used to represent mean and standard deviation, respectively.
## Values in square brackets indicate the 95% confidence interval.
## The confidence interval is a plausible range of population correlations 
## that could have caused the sample correlation (Cumming, 2014).
##  * indicates p < .05. ** indicates p < .01.
##
```

## 1.2 Differences between villages

Comparing body size preferences across villages, we see no significant differences. For body shape, women in Village 3 (low media Mestizo) created bodies with smaller hips compared to waists and busts. Women in Village 1 (high media Creole) also created bodies with smaller waists relative to busts compared to women in the other villages. Note Study 1 ANCOVA and correlation analyses reported in paper were run by TT in SPSS. R versions of analyses by LB are included here for completeness.

```
dazdat1$Village <- relevel(dazdat1$Village, ref = 2)

dazm1 <- lm(daz_BMI ~ Village + Age, data=dazdat1) 
dazm2 <- lm(daz_WHR ~ Village + Age, data=dazdat1)
dazm3 <- lm(daz_WBR ~ Village + Age, data=dazdat1)
dazm4 <- lm(daz_BHR ~ Village + Age, data=dazdat1)

stargazer(dazm1,dazm2,dazm3,dazm4, type = "text")
```

```
## 
## ==================================================================
##                                       Dependent variable:         
##                               ------------------------------------
##                                daz_BMI  daz_WHR  daz_WBR  daz_BHR 
##                                  (1)      (2)      (3)      (4)   
## ------------------------------------------------------------------
## Village1                       -1.803*  0.050***  0.021   0.035** 
##                                (0.935)  (0.016)  (0.015)  (0.014) 
##                                                                   
## Village3                       -1.686*   -0.013  -0.030**  0.018  
##                                (0.868)  (0.015)  (0.014)  (0.013) 
##                                                                   
## Age                             0.128    -0.001   0.001   -0.002* 
##                                (0.087)  (0.002)  (0.001)  (0.001) 
##                                                                   
## Constant                      19.341*** 0.688*** 0.769*** 0.900***
##                                (1.734)  (0.030)  (0.028)  (0.026) 
##                                                                   
## ------------------------------------------------------------------
## Observations                     71        71       71       71   
## R2                              0.104    0.193    0.203    0.103  
## Adjusted R2                     0.064    0.156    0.167    0.063  
## Residual Std. Error (df = 67)   2.917    0.051    0.047    0.044  
## F Statistic (df = 3; 67)       2.594*   5.325*** 5.673***  2.557* 
## ==================================================================
## Note:                                  *p<0.1; **p<0.05; ***p<0.01
```

```
village_proportions <- dazdat1 %>% group_by(Village) %>%
  summarise('Ideal BMI' = mean(daz_BMI, na.rm=TRUE), 
            'Real BMI' = mean(BMI, na.rm=TRUE),
            'Ideal WHR' = mean(daz_WHR, na.rm=TRUE), 
            'Real WHR' = mean(WHR, na.rm=TRUE), 
            'Ideal WBR' = mean(daz_WBR, na.rm=TRUE), 
            'Real WBR' = mean(WBR, na.rm=TRUE), 
            'Ideal BHR' = mean(daz_BHR, na.rm=TRUE), 
            'Real BHR' = mean(BHR, na.rm=TRUE))

as.data.frame(village_proportions)
```

```
#make data long and do violin plots on proportions

f1.1 <- dazdat1 %>% select(ID, Village,WHR, BHR, WBR, daz_WHR, daz_WBR, daz_BHR) %>%
  rename(Real_WHR = WHR,
         Real_WBR = WBR,
         Real_BHR = BHR) %>%
  pivot_longer(3:8, values_to = "Ratio") %>%
  separate(name, c("Type", "Dimension"),sep="_") %>%
  ggplot(aes(Village, Ratio,fill = Type)) + geom_violin() + facet_wrap("Dimension") +
  scale_fill_brewer() + theme_minimal() + theme(legend.position="none") 

f1.2 <- dazdat1 %>% select(ID, Village, BMI, daz_BMI) %>%
  rename(Real_BMI = BMI) %>%
  pivot_longer(3:4, values_to="BMI") %>%
  separate(name, c("Type", "Dimension"),sep="_")%>%
  ggplot(aes(Village, BMI,fill = Type)) + geom_violin()  +
  scale_fill_brewer(labels = c("Ideal","Real")) + theme_minimal()

Figure1 <- cowplot::plot_grid(f1.1, f1.2, ncol = 1)
cowplot::save_plot(Figure1, file="Figure1.jpg")

Figure1
```

## 1.3 Regressions controlling for age

Despite zero order correlations above, once we control for age, overall TV consumption no longer relates to body shape variables although reported frequency of US tv consumption does.

```
#checking all TV hours associations
dazmtv1 <- lm(daz_BMI ~ TVhrs + Age, data=dazdat1) 
dazmtv2 <- lm(daz_WHR ~ TVhrs + Age, data=dazdat1)
dazmtv3 <- lm(daz_WBR ~ TVhrs + Age, data=dazdat1)
dazmtv4 <- lm(daz_BHR ~ TVhrs + Age, data=dazdat1)

stargazer(dazmtv1,dazmtv2,dazmtv3,dazmtv4, type = "text")
```

```
## 
## ==================================================================
##                                       Dependent variable:         
##                               ------------------------------------
##                                daz_BMI  daz_WHR  daz_WBR  daz_BHR 
##                                  (1)      (2)      (3)      (4)   
## ------------------------------------------------------------------
## TVhrs                           0.023    -0.001  -0.001*  -0.0001 
##                                (0.036)  (0.001)  (0.001)  (0.001) 
##                                                                   
## Age                             0.118    0.001    0.002*   -0.001 
##                                (0.078)  (0.001)  (0.001)  (0.001) 
##                                                                   
## Constant                      18.172*** 0.666*** 0.749*** 0.896***
##                                (1.759)  (0.032)  (0.029)  (0.027) 
##                                                                   
## ------------------------------------------------------------------
## Observations                     71        71       71       71   
## R2                              0.033    0.074    0.134    0.015  
## Adjusted R2                     0.004    0.046    0.108    -0.014 
## Residual Std. Error (df = 68)   3.008    0.054    0.049    0.046  
## F Statistic (df = 2; 68)        1.157    2.703*  5.249***  0.518  
## ==================================================================
## Note:                                  *p<0.1; **p<0.05; ***p<0.01
```

```
#checking correlations which were sig with other TV variables

dazmtv2am <- lm(daz_WHR ~ TV_Am + Age, data=dazdat1)
dazmtv3am <- lm(daz_WBR ~ TV_Am + Age, data=dazdat1)
dazmtv1sp <- lm(daz_WBR ~ TV_Sp + Age, data=dazdat1) 
dazmtv2fms <- lm(daz_WBR ~ FILM_Sp + Age, data=dazdat1)
dazmtv3fma <- lm(daz_WBR ~ FILM_Am + Age, data=dazdat1)

stargazer(dazmtv2am,dazmtv3am,dazmtv1sp,dazmtv2fms,dazmtv3fma, type = "text")
```

```
## 
## ==========================================================================
##                                           Dependent variable:             
##                               --------------------------------------------
##                               daz_WHR                daz_WBR              
##                                 (1)      (2)      (3)      (4)      (5)   
## --------------------------------------------------------------------------
## TV_Am                         -0.013** -0.011**                           
##                               (0.005)  (0.005)                            
##                                                                           
## TV_Sp                                            -0.005                   
##                                                 (0.005)                   
##                                                                           
## FILM_Sp                                                  -0.009*          
##                                                          (0.005)          
##                                                                           
## FILM_Am                                                           -0.009* 
##                                                                   (0.005) 
##                                                                           
## Age                            0.0004   0.002   0.003**   0.002*   0.002* 
##                               (0.001)  (0.001)  (0.001)  (0.001)  (0.001) 
##                                                                           
## Constant                      0.699*** 0.772*** 0.747*** 0.754*** 0.755***
##                               (0.036)  (0.033)  (0.035)  (0.031)  (0.031) 
##                                                                           
## --------------------------------------------------------------------------
## Observations                     71       71       71       71       71   
## R2                             0.121    0.159    0.109    0.131    0.133  
## Adjusted R2                    0.095    0.134    0.082    0.106    0.108  
## Residual Std. Error (df = 68)  0.053    0.048    0.050    0.049    0.049  
## F Statistic (df = 2; 68)      4.664**  6.438*** 4.145**  5.138*** 5.219***
## ==========================================================================
## Note:                                          *p<0.1; **p<0.05; ***p<0.01
```

## 1.4 Comparing real and ideal body size/shape

Reviewer 1 requested that we compare real and ideal body dimensions. We find that women’s preferred size and shape differs significantly from actual weigh and shape acros all calculated proportions.

```
t.test(dazdat1$BMI, dazdat1$daz_BMI, paired = TRUE, alternative = "two.sided")
```

```
## 
##  Paired t-test
## 
## data:  dazdat1$BMI and dazdat1$daz_BMI
## t = 7.0919, df = 70, p-value = 8.49e-10
## alternative hypothesis: true difference in means is not equal to 0
## 95 percent confidence interval:
##  2.648874 4.721669
## sample estimates:
## mean of the differences 
##                3.685271
```

```
t.test(dazdat1$WHR, dazdat1$daz_WHR, paired = TRUE, alternative = "two.sided")
```

```
## 
##  Paired t-test
## 
## data:  dazdat1$WHR and dazdat1$daz_WHR
## t = 17.871, df = 70, p-value < 2.2e-16
## alternative hypothesis: true difference in means is not equal to 0
## 95 percent confidence interval:
##  0.1243026 0.1555320
## sample estimates:
## mean of the differences 
##               0.1399173
```

```
t.test(dazdat1$WBR, dazdat1$daz_WBR, paired = TRUE, alternative = "two.sided")
```

```
## 
##  Paired t-test
## 
## data:  dazdat1$WBR and dazdat1$daz_WBR
## t = 4.8623, df = 70, p-value = 6.876e-06
## alternative hypothesis: true difference in means is not equal to 0
## 95 percent confidence interval:
##  0.02141300 0.05119617
## sample estimates:
## mean of the differences 
##              0.03630458
```

```
t.test(dazdat1$BHR, dazdat1$daz_BHR, paired = TRUE, alternative = "two.sided")
```

```
## 
##  Paired t-test
## 
## data:  dazdat1$BHR and dazdat1$daz_BHR
## t = 11.105, df = 70, p-value < 2.2e-16
## alternative hypothesis: true difference in means is not equal to 0
## 95 percent confidence interval:
##  0.07104182 0.10214756
## sample estimates:
## mean of the differences 
##              0.08659469
```

# 2. Study 2 - Body image and eating attitudes

## 2.1 Data correlations

```
corlist <- select(dazdat1, c(BMI, BAStotal, BSQtotal, SATAQtotal, SATAQinternalisation, SATAQpressure, SATAQinformation, EATtotal, TVhrs,   Age, Discrep,daz_BMI))
apa.cor.table(corlist, show.conf.interval=F, filename = "DAZcors1.doc")
```

```
## The ability to suppress reporting of reporting confidence intervals has been deprecated in this version.
## The function argument show.conf.interval will be removed in a later version.
```

```
## 
## 
## Means, standard deviations, and correlations with confidence intervals
##  
## 
##   Variable                M     SD    1           2            3          
##   1. BMI                  24.44 4.83                                      
##                                                                           
##   2. BAStotal             59.42 6.23  .02                                 
##                                       [-.21, .25]                         
##                                                                           
##   3. BSQtotal             15.59 7.81  .48**       -.42**                  
##                                       [.28, .64]  [-.59, -.20]            
##                                                                           
##   4. SATAQtotal           46.54 18.71 .17         -.40**       .41**      
##                                       [-.07, .39] [-.58, -.18] [.20, .59] 
##                                                                           
##   5. SATAQinternalisation 12.13 6.24  .08         -.34**       .38**      
##                                       [-.16, .31] [-.53, -.11] [.16, .56] 
##                                                                           
##   6. SATAQpressure        13.29 7.42  .22         -.41**       .46**      
##                                       [-.02, .43] [-.59, -.20] [.26, .63] 
##                                                                           
##   7. SATAQinformation     13.37 4.21  .10         -.25*        .23        
##                                       [-.14, .33] [-.46, -.02] [-.00, .44]
##                                                                           
##   8. EATtotal             5.40  5.90  .40**       -.29*        .54**      
##                                       [.18, .58]  [-.49, -.06] [.35, .69] 
##                                                                           
##   9. TVhrs                10.98 10.62 .16         -.22         .29*       
##                                       [-.08, .38] [-.43, .01]  [.07, .49] 
##                                                                           
##   10. Age                 19.68 4.96  .22         .30*         -.11       
##                                       [-.02, .43] [.07, .50]   [-.34, .12]
##                                                                           
##   11. Discrep             1.87  5.21  -.03        -.26*        .13        
##                                       [-.27, .21] [-.47, -.03] [-.11, .36]
##                                                                           
##   12. daz_BMI             20.76 3.01  .46**       -.05         .15        
##                                       [.25, .62]  [-.28, .18]  [-.09, .37]
##                                                                           
##   4            5           6            7            8           9           
##                                                                              
##                                                                              
##                                                                              
##                                                                              
##                                                                              
##                                                                              
##                                                                              
##                                                                              
##                                                                              
##                                                                              
##                                                                              
##   .87**                                                                      
##   [.80, .92]                                                                 
##                                                                              
##   .91**        .74**                                                         
##   [.85, .94]   [.61, .83]                                                    
##                                                                              
##   .77**        .53**       .54**                                             
##   [.65, .85]   [.34, .68]  [.35, .69]                                        
##                                                                              
##   .33**        .33**       .39**        .20                                  
##   [.10, .52]   [.10, .52]  [.17, .57]   [-.03, .42]                          
##                                                                              
##   .10          .07         .06          .23          .21                     
##   [-.14, .33]  [-.17, .30] [-.18, .29]  [-.01, .44]  [-.03, .42]             
##                                                                              
##   -.34**       -.19        -.24*        -.47**       -.03        -.37**      
##   [-.53, -.11] [-.40, .05] [-.45, -.00] [-.64, -.26] [-.26, .21] [-.55, -.15]
##                                                                              
##   .09          .01         .07          .11          .06         .15         
##   [-.15, .33]  [-.23, .25] [-.17, .30]  [-.13, .34]  [-.18, .30] [-.10, .37] 
##                                                                              
##   .11          .09         .10          .06          .05         .01         
##   [-.13, .33]  [-.15, .32] [-.14, .32]  [-.18, .29]  [-.19, .28] [-.22, .24] 
##                                                                              
##   10          11          
##                           
##                           
##                           
##                           
##                           
##                           
##                           
##                           
##                           
##                           
##                           
##                           
##                           
##                           
##                           
##                           
##                           
##                           
##                           
##                           
##                           
##                           
##                           
##                           
##                           
##                           
##                           
##                           
##                           
##   -.11                    
##   [-.34, .13]             
##                           
##   .16         -.52**      
##   [-.07, .38] [-.67, -.32]
##                           
## 
## Note. M and SD are used to represent mean and standard deviation, respectively.
## Values in square brackets indicate the 95% confidence interval.
## The confidence interval is a plausible range of population correlations 
## that could have caused the sample correlation (Cumming, 2014).
##  * indicates p < .05. ** indicates p < .01.
##
```

## 2.2 Comparing villages on body image traits

As before, published analyses were run in SPSS but we include linear models for village for completeness. We can see that women in Village 3 (Mestizo, low media) have lower body dissatsifaction than women in the other villages. Women in Village 2 (Mestizo high media) have higher levels of thin ideal internalisation than other women. Older women also show less thin ideal internalisation.

```
mv1 <- lm(BAStotal ~ Age + Village, data=dazdat1)
mv2 <- lm(BSQtotal ~ Age + Village, data=dazdat1)
mv3 <- lm(SATAQtotal ~ Age + Village, data=dazdat1)
mv4 <- lm(EATtotal ~ Age + Village, data=dazdat1)

stargazer(mv1,mv2,mv3,mv4, type="text")
```

```
## 
## ====================================================================================================
##                                                   Dependent variable:                               
##                     --------------------------------------------------------------------------------
##                          BAStotal            BSQtotal            SATAQtotal            EATtotal     
##                             (1)                 (2)                  (3)                 (4)        
## ----------------------------------------------------------------------------------------------------
## Age                       0.316*               0.169              -1.132**              0.158       
##                           (0.180)             (0.225)              (0.485)             (0.173)      
##                                                                                                     
## Village1                   1.967             -5.854**            -15.121***            -4.320**     
##                           (1.931)             (2.416)              (5.257)             (1.878)      
##                                                                                                     
## Village3                   1.152               0.852             -16.103***             -0.930      
##                           (1.791)             (2.241)              (4.833)             (1.727)      
##                                                                                                     
## Constant                 52.209***           13.891***            78.562***             3.960       
##                           (3.581)             (4.479)              (9.661)             (3.452)      
##                                                                                                     
## ----------------------------------------------------------------------------------------------------
## Observations                71                  71                   70                   70        
## R2                         0.105               0.109                0.279               0.075       
## Adjusted R2                0.064               0.069                0.246               0.033       
## Residual Std. Error   6.022 (df = 67)     7.533 (df = 67)     16.247 (df = 66)     5.805 (df = 66)  
## F Statistic         2.607* (df = 3; 67) 2.720* (df = 3; 67) 8.516*** (df = 3; 66) 1.787 (df = 3; 66)
## ====================================================================================================
## Note:                                                                    *p<0.1; **p<0.05; ***p<0.01
```

## 2.3 Testing the sociocultural model.

The model predicts that BMI and internalisation should predict body dissatisfaction and dissatisfaction should in turn predict EAT scores. The relationship between BMI/internalisation and EAT scores should become ns once BSQ is included. This is what we find in regression models. The published paper focuses on mediation models below, but we have the regression models here for completeness and to show that including age and BMI does not change result for mediation models below.

```
#create complete dataset for hierarchical models
dazdat1comp <- subset(dazdat1, EATtotal>-1)

m0 <- lm(BSQtotal~ Age + BMI + SATAQinternalisation, data=dazdat1comp)
m1 <- lm(EATtotal~Age + BMI , data=dazdat1comp)
m2<- lm(EATtotal~Age + BMI + SATAQinternalisation, data=dazdat1comp)
m3<- lm(EATtotal~Age + BMI + SATAQinternalisation+BSQtotal, data=dazdat1comp)


#apa.reg.table(m1,m2,m3,m4)
stargazer(m0,m1,m2,m3, type="text")
```

```
## 
## =============================================================================================================
##                                                        Dependent variable:                                   
##                      ----------------------------------------------------------------------------------------
##                             BSQtotal                                    EATtotal                             
##                               (1)                    (2)                   (3)                   (4)         
## -------------------------------------------------------------------------------------------------------------
## Age                          -0.244                -0.141                -0.068                 0.001        
##                             (0.161)                (0.136)               (0.133)               (0.128)       
##                                                                                                              
## BMI                         0.794***              0.514***              0.470***                0.243        
##                             (0.162)                (0.138)               (0.134)               (0.148)       
##                                                                                                              
## SATAQinternalisation        0.389***                                     0.271**                0.160        
##                             (0.126)                                      (0.104)               (0.105)       
##                                                                                                              
## BSQtotal                                                                                      0.286***       
##                                                                                                (0.096)       
##                                                                                                              
## Constant                     -3.687                -4.392               -8.039**               -6.985*       
##                             (4.808)                (3.868)               (3.962)               (3.761)       
##                                                                                                              
## -------------------------------------------------------------------------------------------------------------
## Observations                   70                    70                    70                    70          
## R2                           0.372                  0.171                 0.249                 0.340        
## Adjusted R2                  0.343                  0.146                 0.215                 0.299        
## Residual Std. Error     6.347 (df = 66)        5.454 (df = 67)       5.230 (df = 66)       4.943 (df = 65)   
## F Statistic          13.028*** (df = 3; 66) 6.921*** (df = 2; 67) 7.305*** (df = 3; 66) 8.355*** (df = 4; 65)
## =============================================================================================================
## Note:                                                                             *p<0.1; **p<0.05; ***p<0.01
```

### 2.3.1 Formal mediation model as published

```
# Doing formal mediation model

# using processR to do it with pretty output

#simple mediation
#pmacroModel(4)
#model list here: http://www.regorz-statistik.de/en/process_3_model_templates.html


labels=list(X="SATAQinternalisation", M="BSQtotal", Y="EATtotal")
process.model <- tripleEquation(X="SATAQinternalisation", M="BSQtotal", Y="EATtotal")
fit3 <- sem(process.model, data = dazdat1)
summary(fit3)
```

```
## lavaan 0.6-8 ended normally after 13 iterations
## 
##   Estimator                                         ML
##   Optimization method                           NLMINB
##   Number of model parameters                         5
##                                                       
##                                                   Used       Total
##   Number of observations                            70          71
##                                                                   
## Model Test User Model:
##                                                       
##   Test statistic                                 0.000
##   Degrees of freedom                                 0
## 
## Parameter Estimates:
## 
##   Standard errors                             Standard
##   Information                                 Expected
##   Information saturated (h1) model          Structured
## 
## Regressions:
##                    Estimate  Std.Err  z-value  P(>|z|)
##   BSQtotal ~                                          
##     SATAQntrnl (a)    0.474    0.139    3.411    0.001
##   EATtotal ~                                          
##     SATAQntrnl (c)    0.137    0.102    1.345    0.178
##     BSQtotal   (b)    0.366    0.081    4.523    0.000
## 
## Variances:
##                    Estimate  Std.Err  z-value  P(>|z|)
##    .BSQtotal         51.859    8.766    5.916    0.000
##    .EATtotal         23.732    4.011    5.916    0.000
## 
## Defined Parameters:
##                    Estimate  Std.Err  z-value  P(>|z|)
##     indirect          0.173    0.064    2.723    0.006
##     direct            0.137    0.102    1.345    0.178
##     total             0.310    0.107    2.900    0.004
##     prop.mediated     0.559    0.222    2.519    0.012
```

```
statisticalDiagram(4, labels=labels,fit=fit3, whatLabel="est")
```

```
AIC(fit3)
```

```
## [1] 905.3771
```

### 2.3.2 Exploratory models on the role of BAS

We can also use BAS and see if positive body image shows the same (inverse) pattern as body disatisfaction measured by the BSQ - but we find that there’s no effect of BAS on EAT once the direct effect of internalisation is accounted for.

```
# Substituting BAS instead of BSQ 


labels=list(X="SATAQinternalisation", M="BAStotal", Y="EATtotal")
process.model <- tripleEquation(X="SATAQinternalisation", M="BAStotal", Y="EATtotal")
fit4 <- sem(process.model, data = dazdat1)
summary(fit4)
```

```
## lavaan 0.6-8 ended normally after 16 iterations
## 
##   Estimator                                         ML
##   Optimization method                           NLMINB
##   Number of model parameters                         5
##                                                       
##                                                   Used       Total
##   Number of observations                            70          71
##                                                                   
## Model Test User Model:
##                                                       
##   Test statistic                                 0.000
##   Degrees of freedom                                 0
## 
## Parameter Estimates:
## 
##   Standard errors                             Standard
##   Information                                 Expected
##   Information saturated (h1) model          Structured
## 
## Regressions:
##                    Estimate  Std.Err  z-value  P(>|z|)
##   BAStotal ~                                          
##     SATAQntrnl (a)   -0.341    0.113   -3.022    0.003
##   EATtotal ~                                          
##     SATAQntrnl (c)    0.245    0.111    2.201    0.028
##     BAStotal   (b)   -0.190    0.111   -1.717    0.086
## 
## Variances:
##                    Estimate  Std.Err  z-value  P(>|z|)
##    .BAStotal         34.257    5.790    5.916    0.000
##    .EATtotal         29.429    4.974    5.916    0.000
## 
## Defined Parameters:
##                    Estimate  Std.Err  z-value  P(>|z|)
##     indirect          0.065    0.043    1.493    0.135
##     direct            0.245    0.111    2.201    0.028
##     total             0.310    0.107    2.900    0.004
##     prop.mediated     0.209    0.151    1.384    0.166
```

```
statisticalDiagram(4, labels=labels,fit=fit4, whatLabel="est")
```

```
labels=list(X="BAStotal", M="BSQtotal", Y="EATtotal")
process.model <- tripleEquation(X="BAStotal", M="BSQtotal", Y="EATtotal")
fit5 <- sem(process.model, data = dazdat1)
summary(fit5)
```

```
## lavaan 0.6-8 ended normally after 13 iterations
## 
##   Estimator                                         ML
##   Optimization method                           NLMINB
##   Number of model parameters                         5
##                                                       
##                                                   Used       Total
##   Number of observations                            70          71
##                                                                   
## Model Test User Model:
##                                                       
##   Test statistic                                 0.000
##   Degrees of freedom                                 0
## 
## Parameter Estimates:
## 
##   Standard errors                             Standard
##   Information                                 Expected
##   Information saturated (h1) model          Structured
## 
## Regressions:
##                    Estimate  Std.Err  z-value  P(>|z|)
##   BSQtotal ~                                          
##     BAStotal   (a)   -0.518    0.136   -3.810    0.000
##   EATtotal ~                                          
##     BAStotal   (c)   -0.075    0.104   -0.726    0.468
##     BSQtotal   (b)    0.382    0.083    4.600    0.000
## 
## Variances:
##                    Estimate  Std.Err  z-value  P(>|z|)
##    .BSQtotal         50.093    8.467    5.916    0.000
##    .EATtotal         24.164    4.084    5.916    0.000
## 
## Defined Parameters:
##                    Estimate  Std.Err  z-value  P(>|z|)
##     indirect         -0.198    0.067   -2.934    0.003
##     direct           -0.075    0.104   -0.726    0.468
##     total            -0.273    0.108   -2.534    0.011
##     prop.mediated     0.724    0.300    2.411    0.016
```

```
statisticalDiagram(4, labels=labels,fit=fit5, whatLabel="est")
```

```
labels=list(X="BAStotal", M="SATAQinternalisation", Y="BSQtotal")
process.model <- tripleEquation(X="BAStotal", M="SATAQinternalisation", Y="BSQtotal")
fit6 <- sem(process.model, data = dazdat1)
summary(fit6)
```

```
## lavaan 0.6-8 ended normally after 16 iterations
## 
##   Estimator                                         ML
##   Optimization method                           NLMINB
##   Number of model parameters                         5
##                                                       
##                                                   Used       Total
##   Number of observations                            70          71
##                                                                   
## Model Test User Model:
##                                                       
##   Test statistic                                 0.000
##   Degrees of freedom                                 0
## 
## Parameter Estimates:
## 
##   Standard errors                             Standard
##   Information                                 Expected
##   Information saturated (h1) model          Structured
## 
## Regressions:
##                          Estimate  Std.Err  z-value  P(>|z|)
##   SATAQinternalisation ~                                    
##     BAStotal   (a)         -0.338    0.112   -3.022    0.003
##   BSQtotal ~                                                
##     BAStotal   (c)         -0.404    0.139   -2.910    0.004
##     SATAQntrnl (b)          0.336    0.140    2.408    0.016
## 
## Variances:
##                    Estimate  Std.Err  z-value  P(>|z|)
##    .SATAQintrnlstn   33.942    5.737    5.916    0.000
##    .BSQtotal         46.261    7.820    5.916    0.000
## 
## Defined Parameters:
##                    Estimate  Std.Err  z-value  P(>|z|)
##     indirect         -0.114    0.060   -1.883    0.060
##     direct           -0.404    0.139   -2.910    0.004
##     total            -0.518    0.136   -3.810    0.000
##     prop.mediated     0.219    0.121    1.817    0.069
```

```
statisticalDiagram(4, labels=labels,fit=fit6, whatLabel="est")
```

```
labels=list(X="TVhrs", M="SATAQinternalisation", Y="BSQtotal")
process.model <- tripleEquation(X="TVhrs", M="SATAQinternalisation", Y="BSQtotal")
fit7 <- sem(process.model, data = dazdat1)
summary(fit7)
```

```
## lavaan 0.6-8 ended normally after 15 iterations
## 
##   Estimator                                         ML
##   Optimization method                           NLMINB
##   Number of model parameters                         5
##                                                       
##                                                   Used       Total
##   Number of observations                            70          71
##                                                                   
## Model Test User Model:
##                                                       
##   Test statistic                                 0.000
##   Degrees of freedom                                 0
## 
## Parameter Estimates:
## 
##   Standard errors                             Standard
##   Information                                 Expected
##   Information saturated (h1) model          Structured
## 
## Regressions:
##                          Estimate  Std.Err  z-value  P(>|z|)
##   SATAQinternalisation ~                                    
##     TVhrs      (a)          0.040    0.070    0.573    0.566
##   BSQtotal ~                                                
##     TVhrs      (c)          0.193    0.079    2.461    0.014
##     SATAQntrnl (b)          0.451    0.134    3.379    0.001
## 
## Variances:
##                    Estimate  Std.Err  z-value  P(>|z|)
##    .SATAQintrnlstn   38.190    6.455    5.916    0.000
##    .BSQtotal         47.728    8.068    5.916    0.000
## 
## Defined Parameters:
##                    Estimate  Std.Err  z-value  P(>|z|)
##     indirect          0.018    0.032    0.565    0.572
##     direct            0.193    0.079    2.461    0.014
##     total             0.212    0.085    2.502    0.012
##     prop.mediated     0.086    0.143    0.601    0.548
```

```
statisticalDiagram(4, labels=labels,fit=fit7, whatLabel="est")
```

## 2.4 SEM with lavaan to investigate the possibility that BAS / SATAQ -> BSQ -> EAT

```
model1 <- ' # direct effects
             EATtotal ~ d*BAStotal + e*SATAQinternalisation
           # mediator
             BSQtotal ~ a*BAStotal + b*SATAQinternalisation
             EATtotal ~ c*BSQtotal
           # indirect effect (a*b)
             abc := a*b*c
           # total effect
             total := d*e + (a*b*c)
         '
fit1 <- sem(model1, data = dazdat1)
summary(fit1)
```

```
## lavaan 0.6-8 ended normally after 14 iterations
## 
##   Estimator                                         ML
##   Optimization method                           NLMINB
##   Number of model parameters                         7
##                                                       
##                                                   Used       Total
##   Number of observations                            70          71
##                                                                   
## Model Test User Model:
##                                                       
##   Test statistic                                 0.000
##   Degrees of freedom                                 0
## 
## Parameter Estimates:
## 
##   Standard errors                             Standard
##   Information                                 Expected
##   Information saturated (h1) model          Structured
## 
## Regressions:
##                    Estimate  Std.Err  z-value  P(>|z|)
##   EATtotal ~                                          
##     BAStotal   (d)   -0.047    0.105   -0.452    0.652
##     SATAQntrnl (e)    0.126    0.104    1.217    0.224
##   BSQtotal ~                                          
##     BAStotal   (a)   -0.404    0.139   -2.910    0.004
##     SATAQntrnl (b)    0.336    0.140    2.408    0.016
##   EATtotal ~                                          
##     BSQtotal   (c)    0.353    0.085    4.130    0.000
## 
## Variances:
##                    Estimate  Std.Err  z-value  P(>|z|)
##    .EATtotal         23.663    4.000    5.916    0.000
##    .BSQtotal         46.261    7.820    5.916    0.000
## 
## Defined Parameters:
##                    Estimate  Std.Err  z-value  P(>|z|)
##     abc              -0.048    0.024   -1.991    0.046
##     total            -0.054    0.025   -2.168    0.030
```

```
AIC(fit1)
```

```
## [1] 901.1773
```

```
lavaanPlot(model = fit1, node_options = list(shape = "box", fontname = 
            "Helvetica"), edge_options = list(color = "grey"), coefs = TRUE,covs=
             TRUE,stars = c("regress"))
```

# 3. Study 3 - Longitudinal sociocultural analyses

## 3.1 Main analyses

```
#center variables
# library(robumeta)
# dazdat$SATAQintern_cent <- group.center(dazdat$SATAQinternalisation, dazdat$ID)
# dazdat$BSQtotal_cent <- group.center(dazdat$BSQtotal, dazdat$ID)

#show how many ppl participated more than once
dazdat1 %>% group_by(times_participated,Village) %>% 
  count()
```

```
#select repeat participants only
dazdatlong <- subset(dazdat, times_participated>1)

#run sequential models for paths in sociocultural model
ml0 <- lmer(BSQtotal~SATAQinternalisation + (1|ID), data=dazdatlong)
ml2<- lmer(EATtotal~SATAQinternalisation+ (1|ID), data=dazdatlong)
ml3<- lmer(EATtotal~SATAQinternalisation+BSQtotal+ (1|ID), data=dazdatlong)
ml4<- lmer(EATtotal~SATAQinternalisation+BSQtotal + TIME + (1|ID), data=dazdatlong)


stargazer(ml0, ml2,ml3,ml4, type="text")
```

```
## 
## =========================================================
##                              Dependent variable:         
##                      ------------------------------------
##                      BSQtotal           EATtotal         
##                         (1)      (2)      (3)      (4)   
## ---------------------------------------------------------
## SATAQinternalisation 0.407***   0.236*   0.054    0.050  
##                       (0.141)  (0.127)  (0.117)  (0.112) 
##                                                          
## BSQtotal                                0.449*** 0.428***
##                                         (0.092)  (0.088) 
##                                                          
## TIMET2                                            -0.077 
##                                                  (1.720) 
##                                                          
## TIMET3                                           3.443***
##                                                  (1.263) 
##                                                          
## Constant             11.502*** 3.842**   -1.328   -2.354 
##                       (1.821)  (1.656)  (1.791)  (1.867) 
##                                                          
## ---------------------------------------------------------
## Observations            76        76       76       76   
## Log Likelihood       -258.344  -250.923 -241.948 -235.370
## Akaike Inf. Crit.     524.687  509.847  493.896  484.740 
## Bayesian Inf. Crit.   534.010  519.170  505.550  501.055 
## =========================================================
## Note:                         *p<0.1; **p<0.05; ***p<0.01
```

## 3.2 Checking basic effects of timepoint

For completeness, we see that Timepoint 3 was also significantly different from Timepoint 1 even without BSQ score and SATAQ in the model.

```
#basic effects of timepoint
mlt1<- lmer(SATAQinternalisation~ TIME + (1|ID), data=dazdatlong)
mlt2<- lmer(BSQtotal~TIME + (1|ID), data=dazdatlong)
mlt3<- lmer(EATtotal~TIME + (1|ID), data=dazdatlong)


stargazer(mlt1,mlt2,mlt3, type="text")
```

```
## 
## ===========================================================
##                               Dependent variable:          
##                     ---------------------------------------
##                     SATAQinternalisation BSQtotal  EATtotal
##                             (1)             (2)      (3)   
## -----------------------------------------------------------
## TIMET2                     -0.771         -1.729    -0.839 
##                           (1.860)         (2.346)  (1.965) 
##                                                            
## TIMET3                     0.598           0.867   3.857***
##                           (1.366)         (1.717)  (1.438) 
##                                                            
## Constant                 11.020***       15.949*** 5.007***
##                           (1.088)         (1.386)  (1.161) 
##                                                            
## -----------------------------------------------------------
## Observations                 76             76        76   
## Log Likelihood            -239.821       -257.299  -244.367
## Akaike Inf. Crit.         489.641         524.598  498.735 
## Bayesian Inf. Crit.       501.295         536.252  510.388 
## ===========================================================
## Note:                           *p<0.1; **p<0.05; ***p<0.01
```

## 3.3 Repeating key models with age added

Reivewer 1 asked if age was important in these analyses. Note age is subsumed entirely within the participant random effect and timepoint fixed effect. We therefore expect suppression effects with timepoint in particular. Note that with age added to final model, neither timepoint nor age is significant.

```
mlt1a<- lmer(SATAQinternalisation~ TIME + Age + (1|ID), data=dazdatlong)
mlt2a<- lmer(BSQtotal~TIME +  Age + (1|ID), data=dazdatlong)
mlt3a<- lmer(EATtotal~TIME +  Age + (1|ID), data=dazdatlong)


stargazer(mlt1a,mlt2a,mlt3a, type="text")
```

```
## 
## ==========================================================
##                              Dependent variable:          
##                     --------------------------------------
##                     SATAQinternalisation BSQtotal EATtotal
##                             (1)            (2)      (3)   
## ----------------------------------------------------------
## TIMET2                     -1.423         -2.876   -1.965 
##                           (1.916)        (2.410)  (2.033) 
##                                                           
## TIMET3                     -0.410         -1.081   1.965  
##                           (1.588)        (1.994)  (1.672) 
##                                                           
## Age                        0.285          0.546*  0.522** 
##                           (0.234)        (0.291)  (0.234) 
##                                                           
## Constant                   5.673          5.699    -4.772 
##                           (4.523)        (5.633)  (4.536) 
##                                                           
## ----------------------------------------------------------
## Observations                 76             76       76   
## Log Likelihood            -239.618       -255.889 -242.601
## Akaike Inf. Crit.         491.235        523.777  497.201 
## Bayesian Inf. Crit.       505.219        537.762  511.186 
## ==========================================================
## Note:                          *p<0.1; **p<0.05; ***p<0.01
```

```
ml0a <- lmer(BSQtotal~SATAQinternalisation + Age + (1|ID), data=dazdatlong)
ml4a<- lmer(EATtotal~SATAQinternalisation+BSQtotal + TIME +Age  + (1|ID), data=dazdatlong)

stargazer(ml0a, ml4a, type="text")
```

```
## 
## =================================================
##                          Dependent variable:     
##                      ----------------------------
##                         BSQtotal      EATtotal   
##                           (1)            (2)     
## -------------------------------------------------
## SATAQinternalisation    0.378***        0.032    
##                         (0.141)        (0.112)   
##                                                  
## BSQtotal                              0.407***   
##                                        (0.090)   
##                                                  
## TIMET2                                 -0.800    
##                                        (1.803)   
##                                                  
## TIMET3                                  2.421    
##                                        (1.472)   
##                                                  
## Age                      0.366          0.293    
##                         (0.232)        (0.211)   
##                                                  
## Constant                 4.280         -7.310*   
##                         (4.888)        (4.047)   
##                                                  
## -------------------------------------------------
## Observations               76            76      
## Log Likelihood          -257.654      -235.087   
## Akaike Inf. Crit.       525.308        486.174   
## Bayesian Inf. Crit.     536.962        504.820   
## =================================================
## Note:                 *p<0.1; **p<0.05; ***p<0.01
```
