# Supplementary material for: Sociocultural drivers of body image and eating disorder risk in rural Nicaraguan women
Source: J Eat Disord. 2022 Sep 6;10:133. doi: 10.1186/s40337-022-00656-0 (PMC9450464; doi:10.1186/s40337-022-00656-0)
Supplement: Supplementary file 2 — Additional file 2: Table S1. Means and standard deviations for sample characteristics, and media viewing variables by village group. Table S2. Pearson’s correlations between disordered eating behaviours (EAT) and predictors based on Stice’s sociocultural model, media internalisation (SATAQ), general body satisfaction (BAS), body shape concerns (BSQ) and own BMI for Mestizo and Creole women. [file 40337_2022_656_MOESM2_ESM.docx]

Sociocultural drivers of body image and eating disorder risk in rural Nicaraguan women

T Thornborrow, E Evans, MJ Tovee, LG Boothroyd

**Supplementary Materials**

Note: this document contains specific SPSS based analyses referred from main text. For other supplementary results, please see accompanying html output file.

**Village differences in demographics and media consumption**

Table S1. Means and standard deviations for sample characteristics, and media viewing variables by village group

|  |  | Village 1  (Low TV) | Village 2  (High TV) | Village 3  (High TV) |  |
| --- | --- | --- | --- | --- | --- |
|  | Number | 19 | 21 | 22 |  |
| Sample | Age (yrs) | 24.3 (6.29) | 19.1 (3.00) | 16.5 (2.28) | ** |
|  | Education (yrs) | 3.1 (3.77) | 8.2 (3.17) | 10.9 (1.81) | ** |
|  | Income US (yearly) | 321 (518.9) | 515 (791.9) | 84 (260.6) | * |
| Media | TVE | 3.1 (6.13) | 13.0 (9.79) | 16.7 (11.29) | ** |
|  | SPTV | 1.6 (1.34) | 3.7 (0.70) | 3.1 (1.15) | ** |
|  | USTV | 1.1 (1.01) | 1.9 (1.39) | 3.3 (0.93) | ** |
|  | SPFM | 1.5 (1.31) | 1.7 (0.86) | 2.4 (1.18) | ** |
|  | USFM | 1.5 (1.31) | 1.7 (0.89) | 2.9 (1.11) | ** |
|  | Music video | 0.1 (0.31) | 0.8 (1.05) | 1.5 (1.33) | ** |
|  | Novela | 0.6 (1.01) | 2.8 (0.65) | 1.7 (1.27) | ** |

* *p* < .05; ** *p* < .01. TVE = average hours television viewing per week; SPTV = frequency of viewing Latin American / Spanish speaking television content; USTV = frequency of viewing U.S-originating / English language television content; SPFM = frequency of viewing Latin / Spanish speaking films; USFM = frequency of viewing U.S / English language films.

**Media exposure variation between communities**

See Table S1 for means of media viewing variables. There was a significant difference in group means for hours of television viewing per week (TVE), *F*(2,59) = 11.110, *p* < .001, partial ƞ^2^ = 0.274. Village 1 watched less TV than both Village 2 and Village 3 (*p*s < .01) who did not differ from each other (*p*s > .05). Village 1 also watched Latin American / Spanish language television shows (SPTV) less often than the other two samples, Welch’s ANOVA, *F*(2,35.42) = 19.238, *p* < .0001, post hoc ps < .05, who did not differ from each other (*p* > .05). Village 1 watched Spanish language films (SPFM) significantly less often than the Village 3 sample only, *F*(2,59) = 4.045, *p* = .023, post hoc *p* < .05. The Village 3 sample, whose native language is English Creole, watched U.S / English language television shows (USTV), Welch’s ANOVA, *F*(2,2,38.01) = 24.169, *p* < .0001, and films (USFM), *F*(2,59) = 9.878, *p* < .0001, post hoc *p*s < .01, more frequently than both Mestizo groups who did not differ significantly from each other (*p*s > .05). Village 1 also watched music video less frequently than Villages 2 and 3, Welch’s ANOVA, *F*(2,31.25) = 13.842, *p* < .0001, Games-Howell post hoc *p*s < .05, who did not differ from each other (*p* > .05). Novela viewing differed significantly between all three groups, with Village 2 watching the most, followed by Village 3, and Village 1 watching the least, Welch’s ANOVA, *F*(2,36.09) = 33.986, *p* < .0001, Games Howell post hoc *p*s < .01.

**Eating attitude and body image associations within ethnic groups**

Pearson’s correlations were carried out separately for Mestizo and Creole participants to explore possible ethnic differences in predictors of disordered eating behaviours. Among Mestizo women, the same pattern of associations was significant. Among Creole women, however, participants’ own BMI and media internalisation were not significantly associated with disordered eating behaviours. Due to the small sample sizes and the exploratory nature of this part of the analyses, further regression analyses were not carried out.

Table S2. Pearson’s correlations between disordered eating behaviours (EAT) and predictors based on Stice’s sociocultural model, media internalisation (SATAQ), general body satisfaction (BAS), body shape concerns (BSQ) and own BMI for Mestizo and Creole women

|  | 1 | 2 | 3 | 4 | 5 |
| --- | --- | --- | --- | --- | --- |
| 1 EAT |  | .517** | .372* | .531** | -.275 |
| 2 BMI | .059 |  | .200 | .576** | .078 |
| 3 SATAQ | .383 | -.054 |  | .579** | .-.378* |
| 4 BSQ | .637** | .534* | .579** |  | -.212 |
| 5 BAS | -.500* | -.208 | -.658** | -.759** |  |

** p <.05; ** p < .005. Top half of table shows correlations for 40 Mestizo participants, the bottom half shows correlations for 21 Creole participants.*
